# The feasibility of recruiting and retaining men who have sex with men and transgender women in a multinational prospective HIV prevention research cohort study in sub‐Saharan Africa (HPTN 075)

**DOI:** 10.1002/jia2.25600

**Published:** 2020-10-01

**Authors:** Theodorus GM Sandfort, Erica L Hamilton, Anita Marais, Xu Guo, Jeremy Sugarman, Ying Q Chen, Vanessa Cummings, Sufia Dadabhai, Karen Dominguez, Ravindre Panchia, David Schnabel, Fatima Zulu, Doerieyah Reynolds, Oscar Radebe, Calvin Mbeda, Dunker Kamba, Brian Kanyemba, Arthur Ogendo, Michael Stirratt, Wairimu Chege, Jonathan Lucas, Maria Fawzy, Laura A McKinstry, Susan H Eshleman

**Affiliations:** ^1^ HIV Center for Clinical and Behavioral Studies New York State Psychiatric Institute and Columbia University New York NY USA; ^2^ Science Facilitation Department FHI 360 Durham NC USA; ^3^ Perinatal HIV Research Unit University of the Witwatersrand Soweto HPTN CRS Soweto South Africa; ^4^ Vaccine and Infectious Disease Division Fred Hutchinson Cancer Research Center Seattle WA USA; ^5^ Berman Institute of Bioethics Johns Hopkins University Baltimore MD USA; ^6^ Department of Pathology Johns Hopkins University School of Medicine Baltimore MD USA; ^7^ Department of Epidemiology Johns Hopkins Bloomberg School of Public Health Blantyre Malawi; ^8^ Desmond Tutu HIV Centre UCT Medical School Cape Town South Africa; ^9^ Kenya Medical Research Institute (KEMRI) CDC Kisumu Kenya; ^10^ Anova Health Institute Johannesburg South Africa; ^11^ Centre for the Development of People (CEDEP) Blantyre Malawi; ^12^ Division of AIDS Research National Institute of Mental Health Bethesda MD USA; ^13^ Division of AID National Institute of Allergy and Infectious Disease National Institutes of Health Bethesda MD USA; ^14^ FHI 360 Durham NC USA

**Keywords:** HIV, closed cohort study, men who have sex with men, transgender women, sub‐Saharan Africa

## Abstract

**Introduction:**

Men who have sex with men (MSM) and transgender women (TGW) in sub‐Saharan Africa (SSA) are profoundly affected by HIV with high HIV prevalence and incidence. This population also faces strong social stigma and legal barriers, potentially impeding participation in research. To date, few multi‐country longitudinal HIV research studies with MSM/TGW have been conducted in SSA. Primary objective of the HIV Prevention Trials Network (HPTN) 075 study was to assess feasibility of recruiting and retaining a multinational prospective cohort of MSM/TGW in SSA for HIV prevention research.

**Methods:**

HPTN 075, conducted from 2015 to 2017, was designed to enroll 400 MSM/TGW at four sites in SSA (100 per site: Kisumu, Kenya; Blantyre, Malawi; Cape Town, South Africa; and Soweto, South Africa). The number of HIV‐positive persons was capped at 20 per site; HIV‐positive persons already in care were excluded from participation. The one‐year study included five biobehavioural assessments. Community‐based input and risk mitigation protocols were included in study design and conduct.

**Results:**

Of 624 persons screened, 401 were enrolled. One in five participants was classified as transgender. Main reasons for ineligibility included: (a) being HIV positive after the cap was reached (29.6%); (b) not reporting anal intercourse with a man in the preceding three months (20.6%); and (c) being HIV positive and already in care (17.5%). Five (1.2%) participants died during the study (unrelated to study participation). 92.9% of the eligible participants (368/396) completed the final study visit and 86.1% participated in all visits. The main, overlapping reasons for early termination included being (a) unable to adhere to the visit schedule, predominantly because of relocation (46.4%), and (b) unable to contact the participant (32.1%). Participants reported strong motivation to participate and few participation barriers. Four participants reported social harms (loss of confidentiality and sexual harassment by study staff) that were successfully addressed.

**Conclusions:**

HPTN 075 successfully enrolled a multinational sample of MSM/TGW in SSA in a prospective HIV prevention research study with a high retention rate and few documented social harms. This supports the feasibility of conducting large‐scale research trials in this population to address its urgent, unmet HIV prevention needs.

## 1 INTRODUCTION

In sub‐Saharan Africa (SSA), there is increasing recognition of the HIV burden among men who have sex with men (MSM) and transgender women (TGW) and their role in the epidemic. Earlier HIV research and public health efforts in SAA have focused on heterosexual transmission, since that is the main mode of HIV transmission this region [1, 2]. However, multiple epidemiologic studies now show that gay, bisexual and other MSM in SSA are profoundly affected by HIV. The first HIV prevalence study among MSM, conducted in 2004 in Senegal, reported a prevalence of 21.5% [3]; pioneering work with MSM has also been conducted in Kenya [4]. The observed HIV prevalence among MSM in SSA ranges from 4.1% to 49.5% [5, 6, 7, 8, 9]. A systematic review in 2012 estimated the overall HIV prevalence among MSM in SSA to be 18% [10]. A more recent review [11] showed that HIV testing among MSM in SSA has significantly increased over time. However, HIV status awareness is still low, ranging from 6.7% in countries with the most severe legislation against the lesbian, gay, bisexual and transgender communities, to 22.0% in countries with the least severe legislation.

Prevention trials are urgently needed to evaluate HIV prevention interventions among MSM/TGW in SSA [12]. However, more information is needed about the feasibility of conducting such trials in this population. Since the early 2000s, several studies have been conducted among MSM in SSA, demonstrating the feasibility of recruiting this population; however, most of these studies had a cross‐sectional design (e.g. [13, 14]). The limited number of longitudinal cohort studies conducted generally included open cohorts at single sites or in individual countries (e.g. [15, 16]). Less is known about the feasibility of retaining MSM in a multi‐national prospective cohort over time (prior multi‐country longitudinal studies with MSM in SSA were limited to six‐month follow‐up [17]). Information is also needed about the feasibility of achieving optimal adherence to study visits and preventing social harms in this population.

Maintaining a cohort of MSM/TGW in SSA for HIV prevention research could be challenging for several reasons, including physical, social and legal risks that are likely to interfere with study retention. Same‐sex behaviour is criminalized in most SSA countries, with sentences ranging up to the death penalty [18]. Although enforcement of these laws varies by country, participation in MSM/TGW research could imply disclosure of illegal behaviour and could thus have legal repercussions. Also, compared to other parts of the world, countries in SSA are among the least accepting of same‐sex sexuality [19]. Experiences with homophobia, including violence and blackmail, are well‐documented in this population [20, 21]. Recruiting and retaining MSM/TGW, especially for studies in a medical context, also require gaining trust. Prior discriminatory experiences in medical settings may lead MSM/TGW to fear insensitive treatment by study staff and inappropriate disclosure of sexual practices or HIV status [22, 23].

There are also risks for research staff and study integrity. For example, being associated with a study of MSM/TGW might be interpreted as condoning or promoting same‐sex sexuality. There is also the possibility of physical attacks at study sites and negative media coverage of the study or the study population. In other studies of MSM in SSA, offices have been attacked by community members and staff arrested, with the allegation that same‐sex sexual activities were being promoted and that young people were being recruited to become MSM [24]. These types of social harms have not been systematically studied.

In this context, the HIV Prevention Trials Network (HPTN) initiated the HPTN 075 study, with the objective to assess the feasibility of recruiting and retaining 400 MSM/TGW in a multinational prospective cohort [25]. Although the primary focus of HPTN 075 was on MSM, TGW were not excluded because some TGW in SSA socialize and identify with MSM or identify as “gay.” [7, 26] This report describes the preparation of the HPTN 075 study sites for study implementation, recruitment methods and results, retention of study participants and occurrence of social harms.

## 2 METHODS

### 2.1 Study design and population

Study participation included biobehavioural assessments at screening and at five subsequent study visits over one year. Four sites participated: Kisumu, Kenya; Blantyre, Malawi; and Cape Town and Soweto, South Africa. HIV‐positive and HIV‐negative persons were eligible to enrol; the number of HIV‐positive persons was capped at 20 per site. Although TGW could participate, there were no specific efforts to recruit them. The same considerations applied to male sex workers. Screening and enrolment started in June 2015 and ended in July 2016. Data collection ended in July 2017.

### 2.2 Eligibility criteria

Main eligibility criteria included: (a) assigned male sex at birth; (b) 18 to 44 years old; (c) reporting at least one act of anal intercourse in the previous three months with a person reported by the participant to be biologically male; (e) three concordant HIV test results at screening and (d) willing to undergo HIV testing throughout the study and receive test results. An optimal evaluation of the study aim would require a sample of persons who were naïve to HIV research. For that reason, persons who previously participated in a biomedical and/or behavioural intervention or cohort study for HIV or sexually transmitted infections (STIs) were excluded; co‐enrolment in such studies was not permitted. The study protocol was amended to ensure that participants would have access to oral pre‐exposure prophylaxis (PrEP) when it became accessible at some sites (e.g. through demonstration projects); although several PrEP referrals were made, no participants reported initiating PrEP. To evaluate uptake of care, HIV‐positive participants who reported already being in HIV care or on antiretroviral treatment (ART) were also excluded from study participation.

### 2.3 Procedures

Given the potential risks associated with the study, each study site was instructed to develop a site‐specific risk‐mitigation plan (RMP; Appendix S1), guided by international guidelines for HIV prevention trials [27, 28], research with MSM in rights‐constrained environments [29] and ethical guidance from the HPTN [30]. This approach was intended to help both researchers and community organizations safely conduct meaningful research in challenging social, political and human rights contexts; this included use of a checklist of factors to be considered in the design, conduct, and implementation of the study.

Preparation of RMPs included: (a) establishing ongoing engagement with the MSM community and local MSM organizations; (b) building rapport and support with the general community, including health authorities, media, religious leaders and local police; (c) creating a Community Advisory Board (CAB); (d) forming a Protocol Advisory Committee (PAC) for active oversight of study implementation that included members of the MSM community and persons with direct expertise on MSM issues; (e) developing procedures for ensuring study participants’ confidentiality, that included a requirement for staff to sign confidentiality agreements and interact with participants in a non‐judgemental, MSM‐affirming way; (f) sensitizing relevant stakeholders, including study staff, and informing them that disclosing any information about participants was subject to disciplinary measures, up to termination; (g) developing procedures for responding to problems and establishing an emergency committee to facilitate a direct response to any urgent situations, including a communication plan; and (h) systematically assessing possible social harms at study visits, developing a priori responses for addressing such harms and training the study staff on the collection of social harm data and reporting. The preparation of these RMPs likely facilitated the process of obtaining in‐country ethics approval for the study; the study’s focus did not cause any difficulties.

HPTN 075 aimed to recruit a diverse sample of MSM at high risk of acquiring HIV infection. In consultation with the community, each site developed site‐specific strategies to promote study awareness and acceptability and recruitment. This approach (a) allowed for optimal use of the local community’s expertise and customization to local circumstances; and (b) made it easier to adjust strategies if recruitment outcomes lagged at specific sites. Various recruitment strategies were implemented: (a) peer outreach: MSM, hired and trained as peer‐outreach workers (from one to eight per site) who approached potential study participants based on their personal knowledge of and connections to the MSM population; (b) participant referral: participants were asked to refer friends for participation in the study (not incentivized); (c) informational sessions about the study; (d) key informant referral: trusted persons with access to MSM networks distributed study information and encouraged MSM to participate; and (e) indirect recruitment: distribution of announcements via in person and web‐based “gay” venues and events.

Screening for HPTN 075 included administrative procedures, collection of biological samples and HIV and STI testing. Eligible persons who consented to participate subsequently had an enrolment visit and follow‐up visits at weeks 13, 26, 39 and 52. All study visits included structured behavioural assessments, HIV risk reduction counselling, assessment of social impacts, collection of biological samples, HIV testing (if HIV negative at the prior visit) and medical examinations. STI treatment was provided; some participants, if so desired, were referred to a clinic of their choice; one site offered treatment to participants’ sexual partners. Condoms and lubricants were available at each visit. ART adherence assessments and counselling were provided as appropriate. Research participation was incentivized according to local standards (ranging from $4 to $10 US). Participation of employed participants was facilitated by offering flexible appointment times, including in the weekend.

Sites implemented a variety of retention strategies. These included visit reminders (via telephone, text messages, email, Facebook or appointment cards); arrangement of transportation to the study site or reimbursement of transportation costs; home visits based on regularly updated locator information; a welcoming study site environment (courteous treatment by staff; addressing participants by preferred names and pronouns; food and refreshments; magazines, video and Internet access in waiting rooms); free medical services and continued outreach and support through community events, such as educational events, beach days, weekend camps and pageants. Some study participants were not interested in these events due to risk of disclosure.

Behavioural assessments included collection of demographic, behavioural, psychosexual and socioeconomic data, and interest in potential HIV prevention strategies. Evaluation of study participation included barriers and facilitators to participation, study burden and social harms and benefits from participation. As much as possible, assessment tools were adopted that were successfully used in this population in SSA. Other measures were adapted from existing assessments.

After study completion, information was collected from various stakeholders, including research staff, via questionnaires and in‐person interviews to characterize the process at each site for building stakeholder support and determining ideal recruitment strategies. In these evaluations, the following topics were addressed: MSM community involvement and impact; recruitment and retention of participants; ongoing community engagement; study site preparation and implementation; incentives and services; CABs/PACs; emergency committee and future research needs.

### 2.4 Data analysis

Descriptive statistics were used to characterize study recruitment, enrolment and retention, as well as participant demographics, motivation to take part in the study and adverse incidents. Univariate and multivariable logistic regression was used to compare characteristics of participants who completed all study visits with those who did not complete the study or missed one of more visits. A stepwise model was used for multivariable analysis; the significance level for entry and exit of variables in the model was set at 0.3 and 0.35 respectively.

### 2.5 Ethics statement

Study sites received approval from their respective institutional review boards (IRBs) and the Division of AIDS, National Institute of Allergy and Infectious Diseases. Informed consent was obtained separately for screening and enrolment. Participants provided written consent at three sites and oral consent at one site, as directed by the local IRB, because signing a consent form could lead to unintended disclosure.

## 3 RESULTS

**Figure 1:**
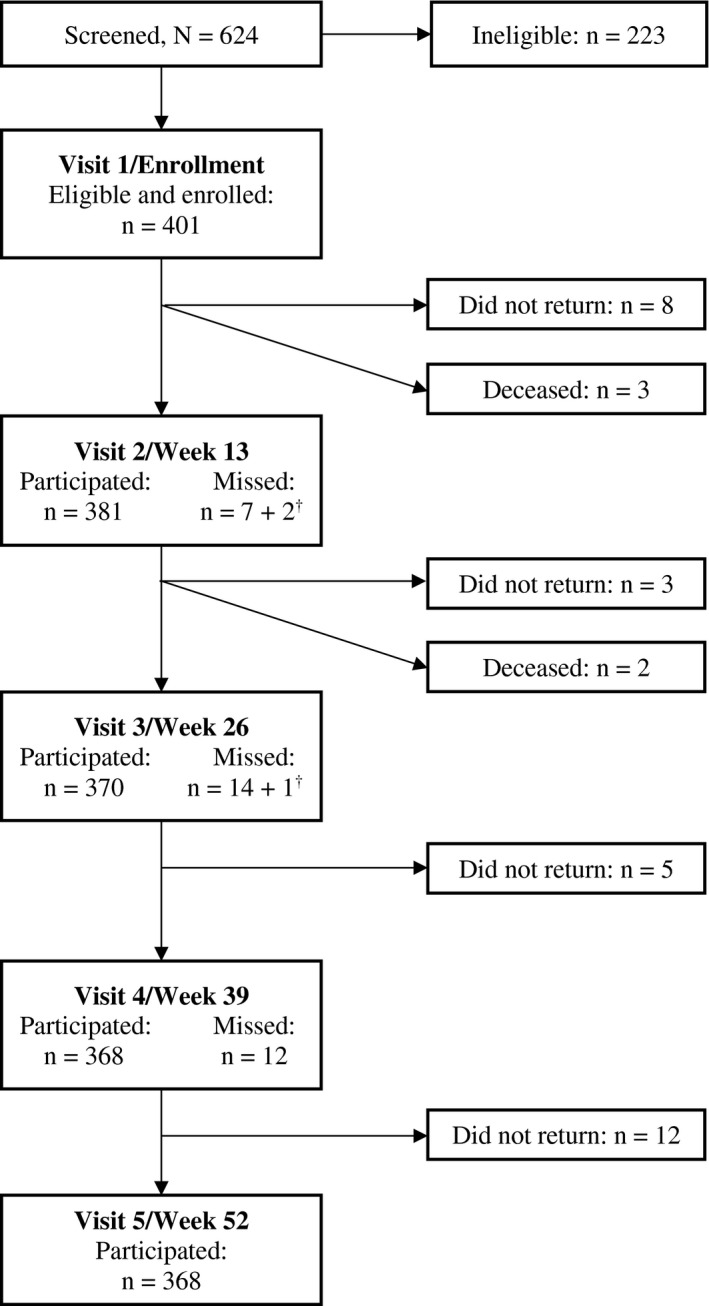
Overview of screening and study participation in HPTN 075. ^†^Visits of men who returned for at least one follow‐up visit but did not complete Visit 5.

A summary of study recruitment and participation outcomes is presented in Figure 1. In total, 624 persons were screened; 223 were ineligible. The main reasons for ineligibility included: (a) being HIV positive after the cap was reached (n = 66, 29.6%); (b) not reporting anal intercourse with a man in the preceding three months (n = 46, 20.6%); (c) being HIV positive and already in care (n = 39, 17.5%); (d) past or current participation in an HIV study (n = 31, 13.9%) and (e) not returning for enrolment within 30 days of the screening visit (n = 29, 13.0%). The time needed to recruit 100 participants varied by site from 18.7 to 39.1 weeks (average 31.1 weeks). The average number of participants recruited per week varied by site from 2.6 to 5.3 (overall average 3.5 per week).

In reviewing the recruitment process, research staff noted the importance of collaborating with local MSM communities and described most of the recruitment strategies as useful and successful. Peer referral was less successful at one site due to stigma and fear of disclosure. Participation incentives attracted persons at some sites who were not MSM, reinforcing the need for rigorous screening. All sites noted the importance of having multiple recruitment strategies. One site observed that specific efforts could have led to a better representation of older and more wealthy participants. A common challenge was the stringent exclusion criterion of being in HIV care, resulting in many persons being excluded (eligibility criteria were not communicated during recruitment or screening; one site made clear that they were looking for persons who were not living with HIV after a substantial number of persons had to be rejected because they were living with HIV). Co‐enrolment in another HIV‐study was observed at one study site where a PrEP demonstration study had started. An attempt to prevent co‐enrolment by jointly introducing biometrics was unsuccessful, because of challenges with obtaining approval from the respective authorities.

### 3.1 Cohort description, motivation to participate and perceived participation barriers

Table 1 presents a description of the 401 men enrolled in the study cohort. The average age was 24.2 years (range 18 to 44 years). Seventy‐one (17.8%) participants tested positive for HIV infection at enrolment (one participant had inconclusive HIV test results). Most participants (62.4%) identified as gay; one in five (20.0%) identified as female or transgender (in line with recommended procedures [31, 32], these persons were categorized as TGW). About two‐thirds of the participants (65.6%) had at least completed secondary education.

**Table 1:** Characteristics of the study cohort (N = 401)

	Kisumu, Kenya (N = 100)	Blantyre, Malawi (N = 100)	Cape Town, South Africa (N = 100)	Soweto, South Africa (N = 101)
	M (median) / n/N^a^ (%)	M (median) / n/N^a^ (%)	M (median) / n/N^a^ (%)	M (median) / n/N^a^ (%)
Age, in years	25.1 (23)	25.2 (24)	23.5 (22)	23.2 (22)
Education				
Grade 11 or lower	33/100 (33.0)	45/96 (46.9)	37/100 (37.0)	27/99 (27.3)
Completed Grade 12	38/100 (38.0)	36/96 (37.5)	39/100 (39.0)	56/99 (56.6)
Completed college	29/100 (29.0)	15/96 (15.6)	24/100 (24.0)	16/99 (16.2)
Married/legal partnership	7/100 (7.0)	10/99 (10.1)	8/100 (8.0)	3/100 (3.0)
Any child	28/100 (28.0)	29/99 (29.3)	20/100 (20.0)	10/100 (10.0)
Transgender^b^	20/99 (20.2)	27/97 (27.8)	18/100 (18.0)	14/99 (14.1)
Sexual attraction				
Men and women	81/100 (81.0)	71/98 (75.4)	36/99 (36.4)	22/100 (22.0)
Men only	19/100 (19.0)	27/98 (27.6)	63/99 (63.6)	78/100 (78.0)
Sexual identity				
Bisexual and other	52/100 (52.0)	50/99 (50.5)	27/100 (27.0)	21/100 (21.0)
Gay	48/100 (48.0)	49/99 (49.5)	73/100 (73.0)	79/100 (79.0)
Ever sex with women	77/100 (77.0)	67/99 (67.7)	50/100 (55.0)	30/100 (30.0)
In ongoing same‐sex,				
Intimate relationship	87/98 (88.8)	90/99 (90.9)	60/95 (63.2)	78/100 (78.0)
HIV positive at screening^c^	15/100 (15.0)	16/99 (16.2)	20/100 (20.0)	20/101 (19.8)

M, mean; n, number with characteristic; N, total number.

^a^ Due to missing values, some n’s do not add up to sample totals

^b^ persons who identified their gender as female or transgender

^c^ the HIV status of one participant could not be determined.

At screening, most participants expressed a strong motivation to participate in the study. Only two participants responded “no” to the question whether, if enrolled, they intended to participate in all scheduled assessments. All participants indicated that it was likely or very likely that they would be able to remain in the study for at least one year. Most participants described themselves as very (81.1%) or moderately (17.0%) committed to this study. In response to the question how important or unimportant participants considered the study to be for their community, the majority said “very important” (88.8%; 9.0% said “moderately important” and 2% “slightly important”). In response to an open question about the single most important reason to participate, one third (33.5%) reported their interest in receiving HIV counselling and testing, and knowing their status. Participants’ answers frequently included more than one reason (Table 2). Most participants (96.5%) felt it was easy or very easy to set up study appointments, make time to come to study visits (91.8%), and travel to the clinic for study visits (89.3%). The distance that participants had to travel ranged from less than a mile up to 20 miles, with travel times ranging from a few minutes to about 60 minutes.

**Table 2:** Most important reason for participating in HPTN 075 (N = 391)^a^

Reason	%	Example
Receiving HIV counselling and testing; knowing one’s status	33.5	“I needed to know about my status”
Receiving HIV risk reduction education	30.9	“I will learn how to keep myself from HIV and get the protective measures and information”
Knowing more about MSM as a community; meeting new people, gaining support from other MSM or being empowered as MSM	20.5	“To get more information about MSM and my lifestyle and challenges that we face as gay people”
Learning more about one’s own health: getting tested for other things than HIV, getting free check‐ups and receiving treatment	16.4	“To pass through the medical tests that would allow me know my health”
Improving one’s general knowledge of health, beyond HIV and STIs	14.8	“To know more about HIV and my health”
Learning about MSM research or contributing to MSM research	10.5	“Because the study involves MSM and I am one of them I think I should participate”

MSM, men who have sex with men; STIs, sexually transmitted infections.

^a^ Based on answers to an open question. Some of the participants’ answers to the open question included more than one reason.

### 3.2 Retention

Five participants (1.2%) died during the study; the causes of death (one sports injury, one case of malaria, one suicide and two murders) were determined by local research staff, after extensive investigation, to be unrelated to study participation. Of the remaining 396 participants, 368 (92.9%) completed the Week 52 Visit and 317 (86.1%) completed all visits; 28 (7.1%) participants did not return after either the Enrolment Visit or any subsequent visit. The main, overlapping reasons for early study termination included: (a) unable to adhere to the visit schedule, predominantly because of relocation (46.4%); (b) unable to contact the participant (32.1%); (c) refusal to participate further (17.9%) and (d) incarceration (3.6%). The proportion of early terminations differed by site, ranging from 0% (Soweto) to 14.0% (Blantyre). A comparison between participants who terminated early and those who participated in all visits, showed that participants in Blantyre and participants with children were more likely to terminate early compared to participants in Kisumu and those without children respectively (Table 3). Study site (Blantyre, compared to Kisumu) was the only factor that remained significant in multivariable analysis. Early termination was not associated with any of the perceptions of the study, including perceived barriers to participation.

**Table 3:** Factors associated with loss to follow‐up during study, HPTN 075 study, Kenya, Malawi, South Africa^a^

	Mean (SD) / n/N (%)		Univariate			Multivariable^b^		
	Completed all visits (N = 341)	Did not complete the study (N = 28)	OR	95% CI	*p* value	AOR	95% CI	*p* value
Country^c^								
Kisumu, Kenya	90/94 (95.7%)	4/94 (4.3%)	REF			REF		
Blantyre, Malawi	84/98 (85.7%)	14/98 (14.3%)	3.73	1.11, 16.17	0.030	3.77	1.12, 16.39	0.029
Cape Town, South Africa	74/84 (88.1%)	10/84 (11.9%)	3.02	0.83, 13.75	0.105	3.29	0.89, 15.09	0.079
Soweto, South Africa	93/93 (100.0%)	0/93 (0.0%)	0.19	0.00, 1.11	0.124	0.22	0.00, 1.32	0.172
HIV status at screening								
Negative	281/304 (92.4%)	23/304 (7.6%)	REF					
Positive	59/64 (92.2%)	5/64 (7.8%)	1.04	0.38, 2.83	0.946			
Age	24.30 (5.50)	25.26 (6.36)	1.03	0.97, 1.10	0.386			
Education								
Low (less than grade 12)	121/132 (91.7%)	11/132 (8.3%)	REF					
Middle (at least grade 12)	144/153 (94.1%)	9/153 (5.9%)	0.69	0.28, 1.71	0.421			
High (beyond secondary school)	71/79 (89.9%)	8/79 (10.1%)	1.24	0.48, 3.23	0.660			
Employment status								
Full or part time employed	102/110 (92.7%)	8/110 (7.3%)	REF					
Self‐employed	45/52 (86.5%)	7/52 (13.5%)	1.98	0.68, 5.80	0.211			
Unemployed (including in‐between jobs)	96/100 (96.0%)	4/100 (4.0%)	0.53	0.15, 1.82	0.314			
Student	86/95 (90.5%)	9/95 (9.5%)	1.33	0.49, 3.61	0.570			
Other	10/10 (100.0%)	0/10 (0.0%)	N.A.					
Marital status								
Single/divorced/widowed	318/342 (93.0%)	24/342 (7.0%)	REF					
Married/civil union/legal partnership	21/25 (84.0%)	4/25 (16.0%)	2.52	0.80, 7.95	0.113			
Any children								
No	268/285 (94.0%)	17/285 (6.0%)	REF			REF		
Yes	71/82 (86.6%)	11/82 (13.4%)	2.44	1.10, 5.45	0.029	2.05	0.87, 5.05	0.139
Transgender								
No	266/289 (92.0%)	23/289 (8.0%)	REF					
Yes	69/74 (93.2%)	5/74 (6.8%)	0.84	0.31, 2.28	0.730			
Sexual attraction								
Men and women	183/200 (91.5%)	17/200 (8.5%)	REF					
Men only	155/166 (93.4%)	11/166 (6.6%)	0.76	0.35, 1.68	0.503			
Sexual identity								
Bisexual and other	133/144 (92.4%)	11/144 (7.6%)	REF					
Gay	206/223 (92.4%)	17/223 (7.6%)	1.00	0.45, 2.20	0.996			
Negative feelings of homosexuality	1.98 (0.54)	1.92 (0.58)	0.79	0.39, 1.59	0.503			
MSM‐related stigma in healthcare	1.84 (0.22)	1.92 (0.20)	9.65	0.84,111.0	0.069			
Concealing same‐sex sexuality	2.16 (1.18)	1.78 (1.16)	0.74	0.52, 1.07	0.113	0.86	0.55, 1.34	0.496
Likelihood to remain in study for a year	1.32 (0.46)	1.22 (0.42)	0.59	0.23, 1.49	0.264			
How committed they felt to participating	1.22 (0.46)	1.28 (0.54)	1.33	0.63, 2.81	0.454			
Importance of study for MSM community	1.14 (0.42)	1.10 (0.32)	0.79	0.27, 2.28	0.658			
Travel to study site	1.92 (0.56)	1.92 (0.54)	1.03	0.52, 2.03	0.942			
Making time for visit	1.90 (0.52)	1.82 (0.54)	0.76	0.36, 1.60	0.470			
Setting up appointment	1.82 (0.48)	1.78 (0.50)	0.89	0.40, 1.99	0.775			

AOR, adjusted odds ratio; CI, confidence intervals; MSM, men who have sex with men; n, number with characteristic; N, total number; OR, odds ratio; REF, reference group; SD, standard deviation.

^a^ Five participants who died during the study were excluded from this table

^b^ Three variables were selected using stepwise model with selection of variables at entry significance level of 0.3 and exit significance level of 0.35

^c^ exact logistic regression analysis is applied due to the zero frequency of participants in the study site Soweto who did not complete the study.

Thirty participants missed a total of 36 visits (including three visits by participants who terminated early). The number of participants who missed visits varied from two to 18 per site. Reasons for missed visits, based on the total number of visits, included (a) unable to schedule a visit, including because of temporary relocation (55.6 %); (b) unable to contact participant (25.0%); (c) refused visit (5.6%); (d) incarcerated (2.8%); (e) other reasons (11.1%). Compared to participants who completed all visits, participants who missed any visit (but completed the Week 52 Visit) were more likely to be younger, to live in Cape Town, to be exclusively attracted to men, to identify as gay and to conceal their sexuality (Table 4). Sexual identity was the only factor that remained a significant predictor of having missed any visits in multivariable analysis. Missed visits were not associated with any perceptions of the study, including perceived barriers to study participation.

**Table 4:** Factors associated with missing one or more study visits, HPTN 075 study, Kenya, Malawi, South Africa^a^

	Mean (SD) / n/N (%)		Univariate			Multivariable^b^		
	Completed all visits (N = 341)	Missed ≥ 1 visits (N = 27)	OR	95% CI	*p* value	AOR	95% CI	*p* value
Country								
Kisumu, Kenya	90/93 (96.8%)	3/93 (3.2%)	REF					
Blantyre, Malawi	84/86 (97.7%)	2/86 (2.3%)	0.71	0.12, 4.38	0.716			
Cape Town, South Africa	74/89 (83.1%)	15/89 (16.9%)	6.08	1.70, 21.81	0.006			
Soweto, South Africa	93/100 (93.0%)	7/100 (7.0%)	2.26	0.57, 9.00	0.248			
HIV status at screening								
Negative	281/302 (93.0%)	21/302 (7.0%)	REF					
Positive	59/65 (90.8%)	6/65 (9.2%)	1.36	0.53, 3.52	0.524			
Age	24.30 (5.50)	21.82 (3.36)	0.88	0.79, 0.98	0.024			
Education								
Low (less than grade 12)	121/129 (93.8%)	8/129 (6.2%)	REF					
Middle (at least grade 12)	144/157 (91.7%)	13/157 (8.3%)	1.37	0.55, 3.40	0.504			
High (beyond secondary school)	71/76 (93.4%)	5/76 (6.6%)	1.07	0.34, 3.38	0.915			
Employment status								
Full or part time employed	102/110 (92.7%)	8/110 (7.3%)	REF					
Self‐employed	45/47 (95.7%)	2/47 (4.3%)	0.57	0.12, 2.78	0.483			
Unemployed (including in‐between jobs)	96/104 (92.3%)	8/104 (7.7%)	1.06	0.38, 2.94	0.907			
Student	86/93 (92.5%)	7/93 (7.5%)	1.04	0.36, 2.98	0.945			
Other	10/12 (83.3%)	2/30 (16.7%)	2.55	0.48, 13.68	0.275			
Marital status								
Single/divorced/widowed	318/344 (92.4%)	26/344 (7.6%)	REF					
Married/civil union/legal partnership	21/22 (95.5%)	1/22 (4.5%)	0.58	0.08, 4.50	0.604			
Any children								
No	268/292 (91.8%)	24/292 (8.2%)	REF					
Yes	71/74 (95.9%)	3/74 (4.1%)	0.47	0.14, 1.61	0.231			
Transgender								
No	266/289 (92.0%)	23/289 (8.0%)	REF					
Yes	69/73 (94.5%)	4/73 (5.5%)	0.67	0.22, 2.00	0.474			
Sexual attraction								
Men and women	183/191 (95.8%)	8/191 (4.2%)	REF					
Men only	155/173 (89.6%)	18/173 (10.4%)	2.66	1.12, 6.28	0.026			
Sexual identity								
Bisexual and other	133/137 (97.1%)	4/137 (2.9%)	REF			REF		
Gay	206/229 (90.0%)	23/229 (10.0%)	3.71	1.26, 10.97	0.018	4.65	1.29, 16.83	0.019
Negative feelings of homosexuality	1.98 (0.54)	2.04 (0.62)	1.19	0.58, 2.46	0.637	1.91	0.80, 4.53	0.144
MSM‐related stigma in healthcare	1.84 (0.22)	1.78 (0.24)	0.31	0.06, 1.49	0.143	0.39	0.07, 2.23	0.288
Concealing same‐sex sexuality	2.16 (1.18)	2.66 (1.26)	1.42	1.01, 1.99	0.045	1.29	0.87, 1.90	0.206
Likelihood to remain in study for a year	1.32 (0.46)	1.30 (0.46)	0.91	0.39, 2.14	0.826			
How committed they felt to participating	1.22 (0.46)	1.08 (0.26)	0.34	0.08, 1.38	0.131			
Importance of study for MSM community	1.14 (0.42)	1.12 (0.42)	0.82	0.29, 2.32	0.703			
Travel to study site	1.92 (0.56)	1.92 (0.48)	1.02	0.50, 2.05	0.962			
Making time for visit	1.90 (0.52)	1.92 (0.54)	1.13	0.52, 2.44	0.756			
Setting up appointment	1.82 (0.48)	1.82 (0.48)	1.01	0.44, 2.32	0.979			

AOR, adjusted odds ratio; CI, confidence intervals; MSM, men who have sex with men; n, number with characteristic; N, total number; OR, odds ratio; REF, reference group; SD, standard deviation.

^a^ Five participants who died during the study were excluded from this table

^b^ four variables were selected using stepwise model with selection of variables at entry significance level of 0.3 and exit significance level of 0.35.

Research staff described the implemented retention activities as effective and necessary. Continuing community involvement helped to promote trust in the study. Interaction with participants and recruiters further offered the opportunity to obtain feedback about the study, and to address concerns and misconceptions (e.g. the misconception that blood draws were used for commercial purposes). Intense mobility in the study population made it hard to reach some participants, despite frequently updating of locator information. In addition, some participants did not have phones, and some lost their phones during the study. A few participants did not have a street address; this required creation of maps to collect locator information, making it harder to locate these participants. Staff reported that some participants provided incorrect locator information because they had not yet disclosed their sexual orientation to their family. Others had concerns that coming to the study site or being seen with other participants might disclose their sexual orientation. School and work obligations made it hard for some participants to meet all appointment times.

### 3.3 Risk mitigation and social harms

Four social harms were reported. Two study participants reported indecent treatment by a male study nurse (inappropriate touching and sexual propositioning). Research staff quickly contacted these participants to address the issue and apologize; staff followed up to help ensure that the participants regained a sense of safety in the study. Site staff were retrained in appropriate behaviour with participants. After a thorough investigation, the nurse involved resigned. Exploration with the site’s outreach workers indicated no negative repercussions in the community. In a different incident, one participant left his job because his employer did not allow him to attend study visits. Finally, one participant reported loss of confidentiality related to being gay; a co‐worker found his informed consent form and told colleagues, which was followed by homophobic comments from his manager. This man considered quitting his job in response. This event resulted in instructing study staff at all sites to be clear to participants about the risks involved in having a signed consent form and more clearly offering the option not to take one’s copy.

After data collection was completed, discussions among research staff indicated that the development of the RMPs sensitized the sites and prepared research staff to deal with a range of problems that might occur. One site commented that preparing the RMP had helped them to focus on dealing with site emergencies more generally.

## 4 DISCUSSION

The HPTN 075 study successfully enrolled a large multinational sample of MSM and TGW in SSA in a prospective HIV prevention research study with high rates of retention and few documented social harms. This indicates that longitudinal research with MSM and TGW in SSA is feasible and can be safely conducted when there is close attention to community engagement and risk mitigation procedures. These results support future efforts to conduct large‐scale HIV prevention research studies and trials with MSM and TGW in SSA to address the urgent and unmet HIV prevention needs in this group.

HPTN 075 represents one of the largest, longest, prospective, multi‐country closed‐cohort research study with MSM and TGW in SSA to date. The study followed 401 men for 12 months, with one screening visit and five study visits. Most prior longitudinal studies of MSM cohorts in SSA were conducted at a single site or in a single country, enrolled open cohorts, or were associated with ongoing clinical care rather than research [33, 34].

The legal status and social marginalization experienced by MSM/TGW in SSA has prompted questions about the feasibility of engaging them in research. The HPTN 075 study had high rates of participant accrual across four sites through a mix of direct and indirect recruitment approaches, including peer outreach, participant and key informant referral, and venue‐based recruitment combined with findings from prior cross‐sectional studies of MSM in SSA [35]. This indicates that it is possible to address recruitment and enrolment challenges in this population.

The HPTN 075 study had high rates of participant retention over one year at four sites in three countries, averaging 92.9%. The high retention was likely driven by the strong commitment and motivation to the study reported by participants. Study retention may also have been enhanced by the novelty of the study and the sense of validation of one’s same‐sex attraction. The relatively high level of education of the participants in HPTN 075 compared to other studies among MSM in SSA (e.g. [33]) might have facilitated participation, even though level of education was not associated with completion of study visits. Participants reported an interest in giving back to their communities through study participation, in addition to receiving key services, including HIV testing and risk reduction counselling; this likely reinforced study participation. The involvement of outreach workers is likely to have facilitated retention. Findings from HPTN 075 further highlight the importance of extensive community and site preparation (amongst others for the delivery of culturally appropriate treatment), active involvement of the community and intense study retention activities. Barriers to retention were limited and were largely related to mobility of participants.

A very important finding in HPTN 075 was that there were very few documented social harms that triggered significant risk mitigation procedures. Given the small number of documented social harms, preparing RMPs might seem superfluous. Alternatively, one could argue that preparation of the RMPs may have reduced the potential for social harms; this was suggested by retrospective discussions with research staff. Without RMPs, staff might have been caught off‐guard and unprepared, which could have aggravated the social harms that occurred. The few social harms that were observed were related to consent procedures and staff training and supervision; future studies should attend closely to these factors.

Some limitations should be considered when evaluating the findings. Because of the study design, it is not possible to state with certainty which factors contributed most to the study’s success. The design also did not allow us to evaluate the efficiency of the various recruitment strategies. Even though the study samples collected at each site were diverse, they are not necessarily representative of the respective populations. Finally, this study was implemented from 2015 to 2017, and although the social situation for sexual minority persons in SSA is not stable, it is extremely likely that what was done to make the study successful is still relevant in the current situation. It is not clear whether COVID‐19 would have an impact on the feasibility of recruitment and retention specific to MSM and TGW.

The results of this study open the door to further large‐scale HIV prevention research with MSM and TGW in SSA. Research is needed to improve understanding of the risks and resiliencies of this key population with respect to HIV transmission, and to develop evidence‐based approaches to meet their urgent HIV prevention needs. MSM and TGW in SSA have previously indicated interest in HIV prevention strategies, including condom use and PrEP [36, 37]. The results of HPTN 075 support the conduct of future trials to advance integrated behavioural and biomedical HIV prevention in these key populations. MSM and TGW in SSA could benefit from inclusion in the next generation of HIV prevention trials to determine whether promising interventions are feasible and effective for this key population, and to facilitate future implementation of HIV prevention interventions in these populations in SSA.

## 5 CONCLUSIONS

Enrolling and retaining MSM and TGW in SSA in a multi‐country, longitudinal, biobehavioural cohort study is feasible and can be conducted safely and successfully. This is especially the case when the local community of MSM and TGW as well as the community more generally are involved in the preparation of the study, and when MSM and TGW play a role in the actual study implementation. Extensive study site preparation seems indispensable. The primary barrier to study participation is the mobility of participants. Retention can be promoted in a variety of ways, including by providing needed services and validation of participants’ sexual minority status. These findings strongly suggest that needed prevention trials with MSM and TGW in SSA are viable.

## COMPETING INTERESTS

Jeremy Sugarman is a member of Merck KGaA’s Bioethics Advisory Panel and Stem Cell Research Oversight Committee, IQVIA’s Ethics Advisory Panel and Aspen Neuroscience's Scientific Advisory Board; he has consulted for Biogen and Portola Pharmaceuticals Inc. None of these relationships are related to the material discussed in this manuscript. None of the other authors has a conflict of interest or a potential conflict of interest to report.

## AUTHORS’ CONTRIBUTIONS

TS, EH, YC, VC, JS, SD, KD, RP, DS, FZ, DR, OR, CM, DK, BK, AO, MS, WC, JL, MF, LM and SE involved in study design and implementation. AM, XG and YC analysed the data. TS, EH and SE wrote the manuscript. EH, AM, XG, VC, JS, SD, KD, DS, MS, WC, JL, YC, MF and LM reviewed manuscript and offered comments and revisions. All authors have read and approved the final manuscript.

## Supporting information


**Appendix S1.** HPTN 075 site‐specific risk mitigation plans.
