# Supplementary material for: The feasibility of recruiting and retaining men who have sex with men and transgender women in a multinational prospective HIV prevention research cohort study in sub‐Saharan Africa (HPTN 075)
Source: J Int AIDS Soc. 2020 Oct 1;23(Suppl 6):e25600. doi: 10.1002/jia2.25600 (PMC7527761; doi:10.1002/jia2.25600)
Supplement: Supplementary file 1 — Appendix S1. HPTN 075 site‐specific risk mitigation plans. [file JIA2-23-e25600-s001.docx]

Blantyre/Malawi Risk Mitigation Plan (Version 0.4)

**HPTN 075 SITE-SPECIFIC RISK MITIGATION PLAN FOR JOHNS HOPKINS RESEARCH PROJECT**

In collaboration with the protocol team, we have developed a site-specific risk mitigation plan, which we will implement as part of the study. This mitigation plan builds upon measures that are incorporated in the protocol, information provided in the site selection questionnaire, and issues that were covered in the provisional site-specific Risk Mitigation Plans. The plan will be reviewed yearly or when there has been new information that has been generated through community and/or stakeholder consultations and also when there is any unexpected policy. This is important in ensuring maximization of participant’s rights, interests and welfare.

**General principles**

We will implement HPTN 075 according to international guidelines for HIV prevention trials (1, 2) and for conducting research with MSM in rights-constrained environments (3), and to the ethical guidance provided by the HIV Prevention Trials Network (HPTN) (4). We are familiar with these guidelines and subscribe to them.

**Ongoing dynamic community consultation**

We will develop an ongoing, dynamic engagement with the MSM community and the broader general community, starting before study implementation. We will document and evaluate implementation of activities to prepare the study site as well as our efforts to optimally engage MSM.

We have made contact with a local organization that works with MSM, namely Center for Development for People (CEDEP), to discuss HPTN 075. Our contact to date has consisted of a continuous discussion on the draft protocol, recruitment strategies, and community involvement. CEDEP has indicated that they are aware of and are supportive of the conduct of the study, including the collaborative mapping of the variety of MSM expressions in the community, development of site-specific strategies for the promotion of study awareness and acceptability in the relevant communities, and development and implementation of confidential recruitment strategies (see Section 3.3 of Protocol). We have a support letter from them attached. We will utilize CEDEP peer educators that are already working on CEDEP’s peer education program to refer potential participants to the study. Those that are enrolled in the study will be asked to let their friends or partners know about the study.

We will foster ongoing consultation with CEDEP over the course of the study by conducting bimonthly meetings, periodic consultations, involvement of representatives on the PAC and we already have a representative on the site CAB. We will also be asking CEDEP to organize MSM so that we can meet them and talk to them about the study or seek their input on the study conduct as direct stakeholders. CEDEP has been active in this community for a number of years and has good relationships with many stakeholders in the Blantyre community.

We expect that this involvement with the community will contribute to the community’s trust in the study and promote study retention. We have a very good and well established relationship with the community since we have been conducting studies from 1989. We will conduct sensitization meetings with different stakeholders throughout the study period to explain the purpose of HIV prevention research and the importance of completing research study visits, and to increase awareness about HIV/AIDS more generally. (See Section 3.6 of Protocol). As part of the development of the recruitment strategies, we will ask community representatives to identify the various MSM groups present in the community. We will use their projections to collaboratively review recruitment outcomes. Through ongoing involvement with CEDEP, we will be able to identify early any potential problems related to recruitment or study participation. Furthermore, these interactions will allow us to explore whether MSM that are recruited for this study represent the MSM present in the various communities, or whether the cohort of recruited men is different in substantive way.

CEDEP will also be involved in the implementation of the monthly off-site peer-education visits for consenting study participants to promote retention and elicit timely feedback regarding study implementation.

**Community Advisory Board and Protocol Advisory Committee**

To facilitate ongoing community engagement, of critical importance for research with the population of MSM in sub-Sahara African settings, we will work with a Community Advisory Board (CAB) and a Protocol Advisory Committee (PAC) (see Section 2.3 of Protocol). The site has an experienced and diversified CAB which consist of community representatives, including government administrators e.g., area chiefs, police officer, religious leaders, health care workers, opinion leaders and a representation of MSM organization. The CAB will advise on general matters of research conduct ensuring that HPTN 075 is being implemented in an ethical manner and that participants are being respected and protected. Since CAB members are community members themselves, they are experts in community involvement. We will also utilize the already available stakeholder structures put in place by CEDEP, a local NGO working with MSM in Malawi.

The PAC will consist of members of the MSM community and others who are most familiar with MSM issues. In case the study elicits negative reactions from the general community, the PAC will consider effective responses and implement those in consultation with the emergency committee (see below) and the site Principal Investigator. The PAC will play a more active role in advising on protocol specific issues affecting participants, and would be key in overseeing the study conduct.

Collectively with the CAB and PAC, we will identify and share concerns and priorities of the communities, explore the study’s potential social impact, and develop a priori responses for addressing any issues. The site CAB is an independent grouping that appreciates that MSM are at a higher risk of contracting HIV than the general population and that we cannot be successful in the HIV fight without involving MSM. A study by CEDEP indicated HIV prevalence of 21% in MSM compared to 10% of the general population hence MSM are listed as a key population in the Malawi HIV National Strategic Plan. Two members from the site have been recognized for the work that they do with MSM, Dr Newton Kumwenda chairs the National HIV Research and Surveillance Technical working group (TWG). This TWG is currently working on the National Strategy for HIV/AIDS research which has identified research among MSM in Malawi as a priority. Ms. Fatima Zulu has recently been appointed as a member of the Country Coordinating Mechanism for the global fund (The Malawi Global Fund Coordinating Committee – MGFCC). Her appointment follows her election by civil society organizations advocating inclusion and protection of sexual minorities in all government HIV/AIDS activities including research. In addition the current National Research agenda (see attachment) clearly identifies research among MSM as one of the vulnerable groups as a priority. Clearly MSM community is high on the national HIV agenda.

The site CAB has been trained on MSM issues and the training will be continuous in order to empower them and ensure their autonomy. They also have their by-laws and roles and responsibilities that guide them in implementing their activities. Representatives from the research staff will participate in the meeting of the CAB, to provide information, answer questions, evaluate the needs of the CAB in addition to its direction and action agenda, and monitor its functioning without interfering with its autonomy.

Both CAB members and study staff will be made aware that while they are working on this study or associated with this study, they might pose a risk in the communities as some community members would think that they are promoting homosexual activities. Therefore the site will encourage staff members and CAB members to report any threats experienced or any signs of violence promptly to the emergency committee. We will utilize CEDEP’s human rights and criminal law expertise and other services that are already put in place by CEDEP if need arises. CAB members and study staff will be advised on the existence of the emergency committee and their contact information will be made available to all study staff and CAB members.

**Emergency committee**

To facilitate a direct response to study participant and staff-related emergencies, we will utilize the existing ‘One Stop Center’ which is within the hospital building and it is open 24 hours. This is a center that provides support services to vulnerable and abused individuals managed by the Ministry of Health and the Police Service. It has a well experienced Social Worker who can ably support our participants as well as staff members if need be. It also has a police officer available 24 hours. We will incorporate the center’s social welfare officer and the Police officer in our emergency committee in addition to the PI, CAB member, and CEDEP Director. (see Section 7.0 of Protocol). The committee will be charged with planning approaches to any emergency situations and facilitating a response should they occur. A study specific standard operating procedure (SOP) will be developed for the site in order to facilitate a direct response to any emergency that may occur. This emergency committee will be charged with deciding on the best course of action to resolve an emergency. Additionally, all study staff will be trained on the implementation of this SOP to ensure its efficient implementation; the training will include a reflection on case scenarios to identify what constitutes an emergency and exercises on how various emergency situations can be handled. Examples of potential emergency situations ~~could~~ include social harm to both study participants and staff , any negative media publicity, or any attempts to interfere with study implementation from the community. Depending on the outcome of the emergency situation, we will also notify and consult with CEDEP. As a mandated organization that protects minority rights for continuation of advocating for MSM rights in the community, we will consult with their criminal lawyer if need be. Contact numbers for the emergency committee members will be included in the informed consent form and on the participant’s appointment card.

Additionally, the National Health Sciences Research and Ethics Committee will be notified within 3 days of any incidence.

**Site preparation**

The study site for HPTN 075 will be established and arranged in a manner consistent with principles of confidentiality and taking into account advice from the CAB and PAC. The study will take place at a tertiary hospital in Blantyre which is a very busy facility with all the health services available at the institution. It is also a training hospital. Apart from our studies there are other research institutions operating at this facility conducting clinical trials in Malaria and other non communicable diseases. We are planning to conduct HPTN 075 in an already existing STI clinic, which is known as “MSM friendly clinic” as it is the only clinic that provides MSM tailored health services. The staff members who run this clinic and will also work on the study, have already been trained by Fenway Institute on MSM health and social issues including stigma and discrimination, sexual identity and coming out, anal sex and common same-sex sexual practices, HIV and STIs, condom and lubricants, mental health, and risk reduction.

In addition, we will prepare study staff to ensure readiness for the culturally-appropriate conduct of the study and availability of culturally-competent care for all participants including men who are identified as HIV-infected at enrollment or during the study. All staff will receive protocol-specific training that will include strategies for minimizing the specific risks involved for participants, the study staff, and for the local MSM communities. During this training, staff will also engage in role-play scenarios where they are in the position of an MSM. This will aid in ensuring that all study staff are informed about the specific risks involved for study participants, study staff, and local MSM communities, and know how to respond to these risks if they occur. This training will take place before the study starts so that study staff members are equipped with knowledge and skills on how to interact with study participants in a non-judgmental, MSM-affirming way (see Section 7.0 of Protocol). Areas covered during the protocol training will include MSM behavior, related local laws, confidentiality and challenges associated with being an MSM. Staff training will be continuous throughout the study period and more training needs will be identified as the study is being implemented.

We will also train research staff on the provision of referrals to counseling and social service support to the One Stop Center. One Stop Center will be trained on their role on the protocol and be treated as our partners. We will receive support from HPTN Operations Center to accomplish this. Throughout the study, research staff, CAB, and PAC will collectively identify and share with the study team concerns and priorities of the communities, explore the study’s social impact, and may develop responses for addressing any issues.

The site will also prepare to protect participants beyond standard procedures after appreciating that participants engage in sexual practices that are in varying degrees socially unacceptable and possibly legally prohibited in some societies and that they are vulnerable to discrimination as part of or as a consequence of study participation. In order to protect study participants and staff, and to secure data safety, all study staff will receive training to interact with study participants in a non-judgmental, MSM-affirming way before the start of the study. There will also be an ongoing, dynamic engagement with the MSM community and the broader general community, starting before study implementation.

**Services**

We will offer study participants at each visit a package of clinical and standard-of-care services as part of their involvement in the study. This will include HIV risk-reduction counseling, and provision of condoms and water-based lubrication (both approved according to guidelines from the World Health Organization). These services will be provided on a one-to-one encounter with the participant and the provider in a private room within the study clinic to ensure confidentiality of the study participants.

**Safety reports**

The Investigator will make safety and progress reports to the IRBs/ECs at least annually and within three months of study termination or completion (see Section 8.0 of Protocol). These reports will include the total number of participants enrolled in the study, the number of participants who completed the study, all changes in the research activity, and all unanticipated problems involving risks to human subjects or others. These reports will include all unanticipated problems involving risks to human subjects or others. We will submit documentation of continuing review to the Division of AIDS (DAIDS) Protocol Registration Office, in accordance with the current DAIDS Protocol Registration Policy and Procedure Manual.

**Social harm assessment and reporting**

The site will collect and report all social harms that are reported to study staff members, using a study-specific incident report form (see Section 5.0 of Protocol). This form will query common and MSM-specific social harms such as altered personal relationships, forced change in housing, and physical violence. The form will also include space for a written narrative to document additional details of any social harm experienced. All research staff will be trained to properly complete the form. As a part of study training, research staff will also be trained on the provision of referrals to the One Stop Center for participants counseling and social service support.

Reports of social harms will be reviewed by the Protocol Chair, DAIDS Medical Officer, Protocol Biostatistician, SDMC Project Manager, and LOC CRM quarterly or more often, if indicated, and reported to the medical officer together with any actions that are taken. Social harms will be summarized and reported to appropriate IRB(s) following IRB guidelines.

**Study monitoring**

On-site study monitoring will be performed in accordance with DAIDS policies.

**Practical measures to mitigate risk**

In addition to the above strategies, we will implement several practical measures to mitigate risks. These include the following:

1. Identification of the study:

To safeguard participants’ confidentiality, the study will not be identified as a study of MSM. Recruitment materials and messaging for the study will be focused on participation in a health and wellness study. We will conduct the Health and Wellness study at the clinic where STI and VCT services are provided. It is in the same space that other studies are also taking place..This will avoid getting them identified as MSM resulting in stigma and providing confidentiality at the same time. The study will be integrated with current HPTN and ACTG studies. Therefore it would be very difficult for a non participant to know who is in the clinic for what service. At our site, all participants are heterosexual men and women. We hope integrating HPTN075 into this clinic will remove the stigma that may be associated with MSM in our setting. We are also planning to schedule their follow up visits sparingly per day in order not have so many of them at a time so that they do not attract other clients’ attention and long waiting time.

2. Screening strategy:

The primary aim is to recruit a cohort of high-risk MSM who would be likely to participate in future intervention studies. We will come up with a selection criteria and screening process in order to identify high risk eligible potential participants. We will recruit from the urban area of Blantyre city within a radius of 8km from the clinic. This will allow easy access to the clinic and most of the participants will be those that already access our STI and HIV services at our health facility.

Recruitment plan will be developed in consultation with CAB, PAC and CEDEP. MSM community mapping data will also contribute to inform our recruitment plan. The screening questionnaire will ask men about their sexual behavior with male and female sexual partners in the past 3 months in addition to several other questions. Potential participants will not be asked to self-identify their sexual orientation. In this way, MSM can be selected for participation through the screening questions.

3. Study participation:

As indicated, the study will take place at a clinic within a tertiary health facility, which is at location that provides participants additional anyonymity since they could have a number of reasons for visiting a public hospital beyond participation in a research study. Therefore it will not be easy for outsiders to know that the study is targeting MSM. Additionally, a number of studies involving men and women who are, and are not, living with HIV are conducted at the site. HPTN 075 participants will use a general waiting area so they are not identified by the use of a distinguished space. All study participants at the site are identified by their participant ID number thereby preventing their name from being disclosed and identifying them to a specific study . HPTN 075 participants will follow the same manner of de-identification. It is however possible that with time outsiders may know through other patients recieving services in the same clinic building due to MSM behaviors.

4. Data management:

Detailed locator information, collected at the study Screening Visit and actively reviewed and updated at each subsequent visit, will be stored separately from other study data and kept in double lockable cabinets. For each study participant, a unique ID will be generated and assigned; there will be no personal identifiers. Only specific study personnel will have access to any hard copies of data and the computer files.

5. Monitoring staff interactions with participants:

Weekly meetings will be held to discuss study progress and any other related issues. In these meetings, the Study team will share their experiences, address concerns, and identify training points. Participants will be advised to contact the PI if they have concerns and contact numbers will be included on their appointment cards as well as ICFs. We are planning to do random chats with participants on how they are being handled at the study clinic and come up with corrective measures for any issues. During the study, if a participant complained about the conduct of study staff, we will sanction staff members concerning their behavior guided by Malawi labor laws. This may include termination of their services. Study staff that will be involved in this study will be warned in advance about these sanctions.

6. Staff’s maintenance of confidentiality

Staff will sign confidentiality statements at recruitment and reminded about the same during weekly protocol meetings regarding sharing any participant information, ensuring the privacy of participants. Staff members that will work on the HPTN 075 study are experienced research nurses that have worked on other network studies before, and are conversant with guiding principles of research ethics and human subjects protection. All study-related information will be stored securely at the study site. All participant information will be stored in locked file cabinets in double lockable room. All laboratory specimens, reports, study data collection, process, and administrative forms will be identified by a coded number only to maintain participant confidentiality. All local databases will be secured with password-protected access systems. Forms, lists, logbooks, appointment books, and any other listings that link participant ID numbers to other identifying information will be stored in a separate, locked file in a double lockable room. Participant’s study information will not be released without the written permission of the participant, except as necessary for monitoring by the NIAID and/or its contractors; representatives of the HPTN LOC, SDMC, and/or LC; other government and regulatory authorities, and/or site IRBs/ECs.

7. Sensitivity training of CAB:

We have recruited a CAB member who is a Project Coordinator for CEDEP. He appreciates MSM issues and he is very dedicated to the MSM welfare as he is an MSM himself. This person will be an available resource to other CAB members and will give CAB an MSM face by sharing his personal experiences as well as MSM’s in general. CAB’s have had intensive trainings to appreciate that MSM are at higher risk of HIV compared to the general population, understanding MSM behaviors, social harms, the study itself and help them understand why HIV prevention technologies are required even in this community. These trainings will continue throughout the study period in order to build capacity of the group as well as the community at large which is very important in facilitating the implementation of future research studies and ensure the strength of programming that might be developed as a consequence of research efforts. CAB members sign confidentiality statements upon recruitment into the CAB; these statements are renewed yearly. Confidentiality will be emphasized during CAB training considering the nature of this study.

8. Complaints about treatment by staff:

Staff members who will work on HPTN 075 have been committed to work with MSM since 2010. Before study initiation, the study staff will go through an intensive HPTN 075 protocol, and GCP and Human Subjects Protection courses and training. During study implementation, the Community Educator will have random chats with participants on how they are being handled at the study clinic and come up with corrective measures for any issues. The Community Educator will also share the summarized information from these chats with the study team during weekly protocol meetings and, if needed, solutions will be developed and agreed upon by the team. Participants will also be encouraged to come to the clinic anytime they have an issue and seek audience with the Study Coordinator or PI. The clinic will have a photo of at least two CAB members posted with their contact information so that participants can contact them if they feel like talking to the CAB instead of study staff. For those participants that would feel that their grievances have not been addressed adequately, they would be encouraged to contact the local IRB, CEDEP or institutions listed in the Informed Consent Form.

9. Response to outside interference:

Malawi has a broadly conservative society that is precipitated by cultural barriers and religious ideologies that deem same sex relationships as immoral. The legal framework criminalises same-sex relationship and the penal code suggests that same-sex relationships are an unnatural offence. It is punishable by imprisonment of up to 14 years, however after the imprisonment of some gay couple and their release in 2010 with the UN intervention, gay men are not prosecuted when they are known, but they are just discriminated against. According to a baseline survey conducted in 2010, 95% reported discrimination and verbal abuse which has decreased tremendously over the years.

HPTN 075 will be conducted at Queen Elizabeth Central Hospital which is a well protected and highly guarded area as it is a tertiary hospital and a university teaching hospital. It has a 24 hour security service and a security alarm system. Each entrance has security guards including the study clinic entrance. All security officers will be sensitized about the study and its possible risks and what to do, or who to inform when they sense potential conflicts.

We do not expect any interference from law enforcers or criminal justice system as MSM is included in the `government‘s HIV strategic plan that it is one of the key populations that we need to target. Malawi has an HIV policy that supports MSM HIV prevention work. For the first time in 30 years the Government through National AIDS commission is funding an MSM peer education program through CEDEP. The college of Medicine, CEDEP and The Johns Hopkins University have conducted studies on MSM for many years with no interference at all. This includes a study while the current government was also in power... Since 2010, we have been holding sensitization meetings with different stakeholders including the Ministry of Justice, the Police Service, The Prison Service, Internal Security, Department of Public Prosecutions etc. All these departments are therefore aware of existence of MSM in Malawi and their human rights. We have been working hand in hand with CEDEP to advocate for MSM rights and creating awareness with these departments to an extent that no action is being taken against any MSM despite that homosexuality remains criminalized. Before the start of the study we will engage with all parties mentioned and educate them about the study.

We have a police officer on our CAB, who will also be a member of the Emergency Committee; he will be contacted in cases of emergencies so that he can easily mobilize his team as quickly as possible, because the police department in Blantyre would have already been made aware of the study after the sensitization meeting that will be held before the study initiation.

In the unexpected case that emergency situations do happen, the emergency committee will meet and discuss dealing with the situation. CEDEP will be contacted immediately for advice, and additional resources such as legal/criminal law expertise. In case the study elicits negative responses in the general community, the PAC will consider effective responses and implement those in consultation with the emergency committee and the site Principal Investigator. The affected individual will be evacuated to a safe place and consider reallocating him/her permanently. A community gate keepers meeting will be held to discuss the incidence and solution to the problem and later a community sensitization meeting will follow. The police, CEDEP and the City Council will be involved in all this.

We do not expect bad media publicity since we have a very good and well established relationship with the media in Malawi since 2003. The media will always check with the site before publishing anything. The media team has already been trained on MSM issues and training will be ongoing and study information will be provided to the team proactively to avoid rumors and misconceptions. If it happens, we will follow a media plan for the study that will contain detailed information on how to release information and how to deal with bad media publicity. The plan will be shared with all parties. We will document frequently asked questions and discuss responses to the questions which may inform our key messages.

**References**

1. Joint United Nations Programme on HIV/AIDS. Good participatory practice. Guidelines for biomedical HIV prevention trials 2011. Geneva: UNAIDS; 2011.
2. Joint United Nations Programme on HIV/AIDS. Ethical considerations in biomedical HIV prevention trials. Geneva: UNAIDS; 2012.
3. amfAR The Foundation for AIDS Research. Respect, protect, fulfill. Best practices guidance in conducting HIV research with gay, bisexual, and other men who have sex with men (MSM) in rights-constrained environments; n.d.
4. HIV Prevention Trials Network Ethics Guidance for Research. HPTN; 2009 available at: http://www.hptn.org/web%20documents/EWG/HPTNEthicsGuidanceV10Jun2009.pdf.

Questions for Researchers to Ask for MSM/HIV Research

| **RESPECT** | Status | Notes |
| --- | --- | --- |
| Have you included the MSM/LGBT community in:  Engagement rules | Yes | As a research site, we have developed a relationship with MSMs in Blantyre since 2010 and we have been providing, HIV prevention information, STI and HIV testing services to this minority group since then. We also have an MSM on our CAB. |
| Situational assessment | Yes | We have partnered with CEDEP, a local NGO that work with Minority populations in Malawi. CEDEP has done studies on MSM including HIV prevalence and their health seeking behavior that we have access to since we are partners. |
| Have you assessed the relevance of the research and potential reactions from greater community structures? | Not yet | We have not reached the greater community yet. However, our CAB is so diversified and we have discussed the relevance of MSM studies with our CAB and we have an idea that the community appreciates the relevance since CAB represents the community well. We have not involved the rest of the community yet because our IRB does not allow that before related studies get approved. |
| Have you assessed the interest amongst the MSM/LGBT community, as well as current infrastructure (or lack thereof)? | Yes | We have done an informal assessment through our chats with MSM that we get in contact with when they come for STI and HIV related services. They have indicated that it is high time that they have their own study specifically targeting their needs. |
| Have you assessed the willingness of your research institution to Respect, Protect, and Fulfill rights of participants? | Yes | The site staff are willing to respect, protect and fulfill rights of participants in MSM studies. |
| Have you developed an MOU with community-based organizations—clearly involving them in all aspects of the research? | Yes | We have an MOU with CEDEP, a local NGO working with minority groups including MSMs. They have assured us of their full support during recruitment and protecting participants’ rights during the study. |
| Have you clearly defined roles and responsibilities of all stakeholders? | Not yet | We have not developed stake holder directory for this study. |
| Have you conducted a comprehensive identification process with stakeholders including:  Community stakeholders, NGOs, CBOs, community groups, informal networks, etc. | Not yet | Our Local IRB will only allow this process after the study approval |
| Government ministries, leaders, etc. |  | Our Local IRB will only allow this process after the study approval |
| Local health care facilities and services |  | Our Local IRB will only allow this process after the study approval |
| Local religious leaders |  | Our Local IRB will only allow this process after the study approval |
| Media |  | Our Local IRB will only allow this process after the study approval |
| Have you engaged government, while first discussing effective models of engagement with community representatives? |  | Our Local IRB will only allow this process after the study approval |
| Have you secured funds for community involvement (e.g., providing financial Incentives, etc.)? | No |  |
| Will you start by conducting formative research activities to learn more about the target populations and their priorities? (This would also include learning about what prior research has been conducted in this population and what are the local perceptions of this research [both from MSM and from non-MSM].) | No | We already have access to study results in this area from CEDEP, our partner. |
| Have you included research on human rights protections/violations within the research context? | No | CEDEP has anecdotal notes on human rights violations. |
| Will you provide research literacy training to key stakeholders?  Local NGOs, CBOs, informal networks of MSM/LGBT | Yes | We will only be able to this after study approval |
| Healthcare service providers |  |  |
| Media |  |  |
| Government |  |  |
| Influential community leaders |  |  |
| **PROTECT** |  |  |
| Have you developed policies for dealing with hostile/intrusive media, media that may blame MSM for ‘spreading HIV’ in a country? | Yes | We have a very good relationship with the media in Malawi since 2003. We have a media team comprising of a representative form each media house in the country. |
| Have you developed certificates of confidentiality to help participants feel safe, knowing that their information will not be shared with others? | Not Yet | Study staff members will sign confidentiality form after they are recruited. Otherwise CAB members already signed a general confidentiality form for all studies. |
| Have you developed personal identifiers that protect people’s identities, or considered conducting research anonymously? |  | Study participants are always identified by Participants Identification Numbers |
| Have you ensured safe storage of any data that would link participants’ sexual orientation information or behavioral practices? |  | Infrastructure and procedures are already in place just like all studies. |
| **FULFILL** |  |  |
| Have you (or others) conducted formative research activities to learn about:  MSM needs and specific priorities | Yes | CEDEP, our partner already has data available. |
| Prior research in this community |  | CEDEP, our partner already has data available. |
| Local perceptions of past research (both from MSM and from non-MSM) |  |  |
| Have you (or others) conducted formative research to learn more about and address structural drivers of HIV and STI risk when researching MSM in low- and middle-income countries? | Yes | CEDEP, our partner already has data available. |
| Criminalization |  |  |
| Stigma and discrimination |  |  |
| Violence/sexual violence |  |  |
| Poverty |  |  |
| Have you planned for MSM/LGBT community capacity-building and informed participation? | Yes | This will be done after study approval |
| Secure funding to build capacity of MSM/LGBT community members | Not yet |  |
| Allow local groups to use resources such as meeting spaces | Yes | MSM hold their HIV sensitization meeting in the auditorium available at the research site. Health workers training too. |
| Ensure representation of MSM/LGBT on staff | Not Yet | To be discussed at site level |
| Train MSM/LGBT community members to be involved as study staff to build capacity for the future |  | To be discussed at site level |
| In disseminating results, do you have plans to work with MSM/LGBT community leaders on data dissemination and a utilization plan, including media advocacy? | Yes |  |
| Do you plan to build the skills of activists to disseminate/use data locally for advocacy? | Yes |  |

Cape Town/South Africa Risk Mitigation Plan (Version 0.4)

**HPTN 075 SITE-SPECIFIC RISK MITIGATION PLAN FOR UNIVERSITY OF CAPE TOWN CLINICAL TRIALS UNIT (UCTCTU) GROOTE SCHUUR HOSPITAL CLINICAL RESEARCH SITE (GSH CRS)**

In collaboration with the protocol team, we have developed a site-specific risk mitigation plan, which we will implement as part of the study. This mitigation plan builds upon measures that are incorporated in the protocol, information provided in the site selection questionnaire, and issues that were covered in the provisional site-specific Risk Mitigation Plans.

**General principles**

We will implement HPTN 075 according to international guidelines for HIV prevention trials (1, 2), for conducting research with MSM in rights-constrained environments (3), and to the ethical guidance provided by the HIV Prevention Trials Network (HPTN) (4). We are familiar with these guidelines and subscribe to them. This risk mitigation plan will be revisited at minimum once per year or more frequently should unexpected changes occur with the study, the site, or local policy. It will also be shared with the site’s IRB and MSM community members for review and additional input.

**Ongoing dynamic community consultation**

The staff at GSH CRS has successfully reached MSM from over 47 distinct communities in the broader Cape Town metro and has facilitated MSM-led community outreach for over seven years. Through these programmes the site has developed numerous strategies for effectively reaching MSM. This prior experience will benefit the study team who will work to recruit MSM from areas where little MSM-focused research has been previously conducted. Buy-in and support from the broader MSM community is at the core of the GSH CRS outreach programming and research philosophy. No work is conducted without their full support and input.

Specifically, the GSH CRS facilitates MSM programming in six township communities with up to 40 MSM actively participating in each township. In each community, weekly discussion groups, social activities and events with other communities are facilitated. These discussion groups create a natural platform to discuss new studies, such as HPTN 075, and offer study staff a meaningful way to engage with MSM in order to listen to their perspectives, concerns, or questions.

Throughout this study, the GSH CRS will use these networks to reassess the perspectives of MSM community members. Given the long-standing relationship between the GSH CRS and many of Cape Town’s MSM communities, there is little concern that MSM will be unsupportive of this study. Historically, MSM community members have been overwhelmingly supportive of MSM focused programs and research, given the lack of MSM programming that is often experienced in Cape Town. Furthermore, meaningful trust has been established between the GSH CRS and MSM community leaders, which we expect will support the study and promote retention.

Specifically, as noted in protocol section 3.3 “Recruitment Process” of the protocol, the GHS CRS will work with MSM to design and implement recruitment strategies for this study. As part of the development of the recruitment strategies, we will consult with community representatives to identify the various MSM groups present in the community. We will use their projections to collaboratively review recruitment outcomes. Through ongoing involvement with MSM in the site’s outreach programme, we will be able to identify early any potential problems related to recruitment or study participation. Furthermore, these interactions will allow us to explore whether MSM that are recruited for this study represent the MSM present in the various communities, or whether the cohort of recruited men is different in substantive way.

The sites’ current outreach activities and networks will also be used in the implementation of the monthly off-site peer-education visits for consenting study participants to promote retention and elicit timely feedback regarding study implementation. The protocol, in section 3.6 “Participant Retention” also highlights multiple methods of participant retention that will be used by the study site. Specifically, participants will be readily engaged throughout their study participation in order to explain the study visit schedule and the importance of individual participation in the study. Additionally, the staff will make immediate follow up with participants after a missed visit by using outreach workers, telephonic and electronic communication, and home visits.

**Community Advisory Board and Protocol Advisory Committee**

As discussed in section 2.3 “Study Design” of the protocol, the site is supported by a functioning and enthusiastic community advisory board (CAB). The CAB is comprised of HIV-positive and HIV-negative individuals who represent the MSM and heterosexual population. CAB participants are initially recruited from individuals participating in studies at the site, who were then requested to invite other community members to join. The CAB’s autonomy is supported by a self-elected executive committee comprising the chair, vice-chair and secretary. This executive committee meets monthly with the study staff (i.e. the CAB liaison) and with the broader CAB (comprising 25 individuals) every second month. Site staff are able to present new and updated study protocols to the CAB for input at each meeting. In order to encourage the autonomy of the CAB, study staff remain present at meetings to answer questions and encourage dialogue, but they do not interfere in discussions or debates among the CAB. Staff are present at each meeting; however, the CAB executive facilitate meetings overall and can request that staff excuse themselves if desired.

Additionally, a committee of MSM community and peer leaders will further support this protocol. These community leaders directly represent many of the communities where recruitment of participants will take place. Each community leader is in direct contact with the project manager and is able to provide direct feedback as to the status of the study’s implementation within their community. Their input and buy in is regularly sought through structured meetings prior to implementation of any activity within each community. Staff members will assist in the facilitation of committee meetings with the peer leaders and, as a means of promoting their autonomy, will continually inform peer leaders that their participation in the committee is voluntary and that it will not impact their participation in other activities with the GSH CRS. Meetings and other engagements are always conducted in safe and private environments. Additionally, committee members are reminder that their participation poses an inherent risk of being identified as an MSM; however, all members are already out and highly visible within their communities.

In case the study elicits negative reactions to the study in the general community, the study staff will consider effective responses and implement those in consultation with the CAB, MSM community leaders and the site Principal Investigator.

Collectively with the CAB and MSM community leaders, we will identify and share concerns and priorities of the communities hosting the research, explore the study’s potential social impact, and develop a priori responses for addressing any issues.

**Emergency committee**

A study specific standard operating procedure (SOP) will be developed by the site in order to facilitate a direct response to study participant- and staff-related emergencies. This SOP will be based on previous emergency response SOPs that already exist at the study site. An emergency committee will be developed and be charged with deciding on the best course of action to resolve the emergency. This committee will include:

- The PI – Prof. Linda-Gail Bekker
- The Project Manager – Mr. Ben Brown
- The Study Coordinator – Mrs. Karen Dominguez

Additionally, two individuals will be considered part of the emergency committee ad hoc, they include:

- The GHS CRS Social Worker
- Communications Director - Lavinia Browne

This provides the emergency committee to remain small and efficient while include the experience and skills such as social work and communication from non-protocol specific individuals.

As noted in section 7.0 of the study protocol, an emergency communication infrastructure will be developed such that the emergency committee is informed immediately upon identification of the emergency. The emergency team will notify the site’s IRB within 3 working days of any emergency situation. Beyond emergency situations, the PI will also make safety and progress reports available to IRB within three months of study termination or completion, as required in section 8.1 of the study protocol.

All staff will receive training on the implementation of this SOP to ensure its efficient implementation. This training will include role-playing scenarios where staff members will practice their responses to key emergency situations. Such exercises will improve all staff members understanding of which scenarios constitutes an emergency, to whom authority is delegated in each of these specific situations, and what key actions should be taken in these emergencies. Examples of potential emergency situations could include any incidences that negatively affect a participant’s wellbeing, any negative publicity about the study in the mainstream media, or any attempts to interfere with study implementation from community-based organizations. In such emergency situations, the team will respond immediately and directly by first engaging with each study participant to inform them of the situation, provide any required support, and ensure their overall wellbeing where possible. Additionally, the emergency team will identify the relevant stakeholders involved in the emergency (i.e. the media, general community members) and establish a direct line of communication with them. Where possible the team will answer questions, address general concerns, and provide education about the study in order to resolve the situation. If needed, the team will also proactively address emergency situations through press releases as well as meetings with community leaders from the general community. An example of a crisis communication template adapted from previous research is attached below.

For participants, an emergency after-hours phone has been set up at the site and is monitored by the study coordinator and project manager for this study. All participants are provided the contact details for the phone should an emergency occur outside of office hours. Additional support and consultation will be provided to the emergency committee through an existing on-call psychologist/social worker. Furthermore, the GSH CRS through partnership with the Sex Workers Advocacy and Education Taskforce (SWEAT) has access to criminal lawyers and police officials should their consultation be necessary.

The constitution of South Africa fully supports the legal rights of the LGBT community, therefore the GSH CFS faces no potential legal repercussions related to conducting research with MSM. Therefore, communication with the South African Department of Justice (DOJ) or South African Police Services (SAPS) would mostly likely only be needed through a standard letter of notification. Additionally, the GHS CRS is an active participant in the South African National AIDS Council (SANAC), which also includes regularly participation from representatives from the DOJ. Should the need arise; staff from the GSH CRS would seek out discussions from the DOJ through pre-existing relationships developed within SANAC.

**Site preparation**

Section 7.0 “Ethical Considerations” identifies the potential risk faced by participants in this study should their confidentiality be breached or they otherwise be exposed as identifying as gay or MSM. This study will be conducted at the University of Cape Town Clinical Trials Unit (UCTCTU) Groote Schuur Hospital Clinical Research Site (GSH CRS), which has multiple measures in place to protect participant confidentiality. First, participant confidentiality is fully supported by the design of the study site. The site is equipped with four counseling rooms, four medical consultation offices, a pharmacy counseling office, two phlebotomy rooms and one procedural room. All clinical and research spaces are private. Additionally, the sites location within a public hospital provides further anonymity to participants. As described in section 8.6 “Confidentiality”, all study-related information is stored within locked storage units located in secured offices. All electronic databases are also password protected and stored on the secured and firewalled GHS CRS server.

Furthermore, as noted in section 7.0 of the study protocol, all study staff are experienced in the culturally appropriate conduct of MSM research and culturally competent care for all participants including men who are identified as HIV-infected at enrollment or during the study. Prior to the study implementation, all staff will receive protocol-specific training that will include strategies for minimizing the specific risks involved for participants, the study staff, and for the local MSM communities. During this training, staff will also engage in role-play scenarios where they are in the position of an MSM. Specific attention will be paid to the need for strict participant confidentiality. As a part of study training, we will also train research staff on the provision of referrals to counseling and social service support. We will receive support from HPTN Operations Center to accomplish this. Staff training will also focus on participant confidentiality. All staff will be held to the GSH CRS human resource policy, which takes a strong stance on participant confidentiality. The policy states that the PI and director of human resources, using the standard HR disciplinary framework, will investigate breaches in participant confidentiality. These investigations will result in immediate termination of the staff member, if found to be responsible. In addition to this standard policy, all staff will be required to sign confidentiality agreements that also reflect this policy and make it clear to staff that they are responsible for upholding participant confidentiality or risk termination. All staff training, particularly concerning participant confidentiality and treatment, will be an ongoing process and refresher trainings will be conducted throughout the course of the study at regularly scheduled staff meetings.

All study staff have previously worked with MSM and are able to interact with study participants in a non-judgmental, MSM-affirming way. All study staff have previously received MSM sensitivity training with topics that include stigma and discrimination, sexual identity and coming out, Anal sex and common sexual practices, HIV and STIs, condom and lubricants, mental health, and risk reduction.

**Services**

We will offer study participants at each visit a package of clinical and standard-of-care services as part of their involvement in the study. The site provides full time employment to 1 medical officer, 4 study coordinators, 3 research nurses and 1 pharmacist. The site is also supported by the part-time employment of 6 medical officers, 1 pharmacist, 2 lab technicians and a psychologist. These trained clinical staff will perform all clinical services including HIV testing, medical examinations, specimen collection and counseling.

The standard services will also include HIV risk-reduction counseling, and the provision of condoms and water-based lubrication. The site currently employees 2 MSM-identified research counselors and 4 MSM-identified outreach workers. Respectively, these staff are fully trained to implement a variety of MSM-specific risk reduction counseling and outreach strategies. Condoms and lubrication will be provided according World Health Organization guidelines and will be locally sourced with input from MSM community members and leaders.

All clinical and counseling services will be provided in a manner to ensure confidentiality of the study participants. For example, condoms and lubricant will be provided in opaque unmarked bags, clinical and counseling services will be provided in private spaces, and all personal identifying information will be removed from study files. Participants will be confidentially referred to one of the site’s MSM-sensitized service providers if they require care or services beyond those offered through the study or at the site.

**Safety reports**

The Investigator will make safety and progress reports to the IRBs/ECs at least annually, and within three months of study termination or completion. These reports will include all unanticipated problems involving risks to human subjects or others (see also below). We will submit documentation of continuing review to the Division of AIDS (DAIDS) Protocol Registration Office, in accordance with the current DAIDS Protocol Registration Policy and Procedure Manual.

**Social harm assessment and reporting**

We will train the staff in the collection and reporting of social harms, as described in section 5.0 “Safety Monitoring and Adverse Event Reporting” of the protocol. Such events will be collected and reported by study staff according to guidelines specified by the protocol team, using a study-specific incident report form, as noted in the protocol. This form will query common and MSM-specific social harms such as altered personal relationships, forced change in housing, and physical violence. The form will also include space for a written narrative to document additional details of any social harm experienced. All research staff will be trained to properly complete the form. As stated in section 5.0 of the study protocol, reports of social harms will be reviewed by the Protocol Chair, DAIDS Medical Officer, Protocol Biostatistician, the Statistical and Data Management Center Project Manager, and Leadership and the Operations Center Clinical Research Manager, quarterly or more often, if indicated, and reported to the Medical Officer together with any actions that are taken. Social harms will be summarized and reported to appropriate IRB(s) following IRB guidelines. Any emergency situations will be reported to the parties mentioned above within 24 hours.

**Study monitoring**

On-site study monitoring will be performed in accordance with DAIDS policies. Additionally, the implementation of this risk mitigation plan will be reviewed externally prior to site activation.

**Practical measures to mitigate risk**

In addition to the above strategies, we will implement several practical measures to mitigate risks. These include the following:

1. Identification of the study:

To safeguard participants’ confidentiality, the study will not be identified as a study of MSM. Instead, the study will be referred to broadly as a men’s health study. To diminish the social harms related to participating in an HIV-related study, all recruitment materials and messaging for the study will focus on participation in a health and wellness study. Recruitment material and study information will not be targeted to the general population or intentionally advertised in public spaces. For example, no large scale poster advertisements will be used in public clinics or meeting spaces. Instead information will be systematically distributed through MSM social networks and in MSM- or LGBT-specific spaces.

Study staff will be trained in strategies to maintain the identification of the study as a men’s health study and they will not disclose the full nature of the study to non MSM-identified community members.

2. Screening strategy:

The screening questionnaire will ask men about their sexual behavior with male and female sexual partners in the past 3 months in addition to several other questions. Potential participants will not be asked to self-identify their sexual orientation. In this way, MSM can be selected for participation through the screening questions.

3. Study participation:

The study site is designed to protect the confidentiality of all participants. Firstly, the study site is located in a large public hospital, Groote Schuur. The location provides participants additional anyonymity since they could have a number of reasons for visiting a public hospital beyond participation in a research study. Additionally, a number of studies involving men and women who are, and are not, living with HIV are conducted at the site. All participants use a general waiting room so they are not identified by the use of a distinguished space. Finally, all participants are identified by their participant ID number thereby preventing their name from being disclosed and identifying them to a specific study.

4. Data management:

Detailed locator information, collected at the study Screening Visit and actively reviewed and updated at each subsequent visit, will be stored separately from other study data and kept in locked cabinets located in locked offices and accessible only to study staff. For each study participant, a unique ID will be generated and assigned; there will be no personal identifiers. Only specific study personnel will have access to any hard copies of data and the computer files.

5. Monitoring staff interactions with participants:

The entire study staff will meet at least once per week to discuss implementation and management of the study. During this meeting any concerns with the conduct of the study can be addressed and corrective action, including staff training can occur. Study participants will engage with a variety of study staff privately during each visit. Each staff will forge an affirming and open relationship with each participant. Staff will then be equipped to share multiple perspectives for each participant during weekly meetings. Should participants have an issue with a staff member they will have the opportunity to discuss that concern with other members of the staff throughout the study visit.

6. Staff’s maintenance of confidentiality:

Staff will sign confidentiality statements at recruitment and on annual basis regarding sharing any participant information, ensuring the privacy of participants. The confidentiality statement will give examples of the kinds of information that should be kept confidential. Additionally, all staff maintain updated certifications in good clinical practice, human subjects protections and good participatory practice.

7. Sensitivity training of CAB:

The site’s CAB has received MSM sensitivity training similar to all study staff members. Prior to the initiation of this study, the CAB will receive refresher MSM sensitivity by an experienced trainer.

8. Complaints about treatment by staff:

In addition to the strategies addressed in point 6, all participants will be provided private ways to inform the PI, project manager, or chairperson of the site IRB of any issues or concerns they have regarding their study participation. Should an issue arise the leadership team of the study will meet to discuss the best course of corrective action to take.

**amfAR Questionnaire:**

Respect

**1. Have you included the MSM/LGBT community in:**

- **Engagement Rules**
- **Situational Assessments**

MSM are involved through every stage of the research process at the GSH CRS from initial community consultations to the study staff themselves. Specifically, MSM taking part in the GSH CRS’s community engagement programme are provided numerous opportunities to lead situational assessments and establish the rules of engagement for new research within their individual communities.

**2. Have you assessed the relevance of the research and potential reactions from greater community structures?**

Feedback has been sought from the MSM community and from the GSH CRS CAB and MSM committee. Relevance and potential reactions from the greater community will take place once the study has been finalized.

**3. Have you assessed the interest amongst the MSM/LGBT community, as well as current infrastructure (or lack thereof)?**

The GHS CRS MSM community engagement programme referenced above provides a specific opportunity for MSM to provide feedback to and engage with research staff. Through this programme, staff are regularly able to assess the interest of MSM for new research initiatives as well as gain understanding of the community infrastructure.

**4. Have you assessed the willingness of your research institutions to Respect, Protect, and Fulfill rights of participants?**

Yes, all staff participating or connected to MSM research have been engaged with appropriate MSM sensitivity training and have confirmed their willingness to respect, protect, and fulfill the rights of all participants.

**5. Have you developed an MOU with community-based organizations – clearly involving them in all aspects of research?**

The GSH CRS MSM research team has engaged community-based organizations since 2008. Initially, MOUs had been developed with key LGBT organizations in order to formalize relationships and responsibilities. Over the years, as the experience of the GSH CRS team grew, these MOU proved no longer necessary. Currently, all main NGOs that contribute to key population-based work (i.e. MSM, sex workers, and transgender individuals) are actively engaged during the launch of new research and throughout its implementation.

**6. Have you clearly defined roles and responsibilities of all stakeholders?**

All key population-based NGOs or community organizations that engage with the MSM research team of the GSH CRS have direct communication access to the PI, Project Manager, and with the research assistant responsible for community engagement. Current roles and responsibilities are focused entirely on sharing of information, facilitation of recruitment, and providing feedback to the research team on their current research initiatives.

**7. Have you conducted a comprehensive identification process with stakeholders including:**

- **Community stakeholders, NGOs, CBOs, community groups, informal networks, etc.**
- **Government ministries, leaders, etc.**
- **Local health care facilities and services**
- **Local religious leaders**
- **Media**

The above stakeholders have been identified and reassessed since exploratory community engagement work in began in 2009.

**8. Have you engaged government, while first discussing effective models of engagement with community representatives?**

Government engagement has occurred mostly through representatives with the South African Department of Health.

**9. Have you secured funds for community involvement (e.g. providing financial incentives, etc.)**

Funding for community involvement occurs through a variety of programmes conducted at the GSH CRS site. All research projects are required to budget for thorough community engagement activities. Financial incentives are not used for standard community engagement activities, however, should participants incur transport costs, they are reimbursed.

**10. Will you start by conducting formative research activities to learn more about the target populations and their priorities? (This would also include learning about what prior research has been conducted in this population and what are the local perceptions of this research [both from MSM and from non-MSM]).**

The GSH CRS team has conducted numerous formative research projects since 2008. These formative research projects have included both focus group discussions and in-depth interviews with MSM and MSM community leaders from distinct communities throughout Cape Town. This research has focused on subjects including: new technology use for HIV prevention, stigma discrimination, access to healthcare services, and community engagement strategies.

**11. Have you included research on human rights protections/violations within the research context?**

Yes, human rights protections and violations were explored during our MSM HIV Surveillance projects in 2009 and 2011.

**12. Will you provide research literacy training to key stakeholders?**

Research literary training has been provided to MSM and participants in the GSH CRS community outreach programme in the form of dialogues and discussion groups specifically focusing on HIV prevention research with MSM.

Protect

**1. Have you developed policies for dealing with hostile/intrusive media, media that may blame MSM for ‘spreading HIV’ in a country?**

Addressing hostile media would fall under the GSH CRS’s standard media and communications policy. This would be addressed by engaging various key stakeholders and partners, such as the Treatment Action Campaign (TAC) who have extensive experience with engaging the media. Additionally, relationships have been built with representatives from the media and these individuals would be engaged directly for consultation.

**2. Have you developed certificates of confidentiality to help participants feel safe knowing that their information will not be shared with others?**

All staff members at the GSH CRS are required to sign confidentiality agreements prior to working participants or participants’ data. Participants are informed of this practice and are able to see the confidentiality agreements should they wish.

**3. Have you developed personal identifiers that protect people’s identities, or considered conducting research anonymously?**

All participant data is de-identified and is not linked to personal identifying information. Participant signatures on informed consent forms are kept separately from files that contact participant data. Although limited, completely anonymous research has been conducted on site previously.

**4. Have you ensured safe storage of any data that would link participants’ sexual orientation information or behavioural practices?**

All participant data is stored securely in locked cupboards and is only accessible by specific staff members.

Fulfill

**1. Have you (or others) conducted formative research activities to learn about:**

- **MSM needs and specific priorities**
- **Prior research in this community**
- **Local perceptions of past research (both from MSM and from non-MSM)**

Yes, as detailed above, numerous formative research studies have been conducted by the GSH CRS team. This research includes both focus groups discussions and in-depth interviews with MSM, MSM community leaders, and other key stakeholders throughout Cape Town. Specific formative research has focused on capacity building and community development through which information regarding previous activities, MSM needs, and perceptions of current and past work was asked of participants.

**2. Have you (or others), conducted formative research to learn more about and address structural drivers of HIV and STI risk when researching MSM in low- and middle-income countries?**

- **Criminalization**
- **Stigma and Discrimination**
- **Violence/Sexual Violence**
- **Poverty**

Yes, as detailed above, numerous formative research studies have been conducted by the GSH CRS team. This research includes both focus groups discussions and in-depth interviews with MSM, MSM community leaders, and other key stakeholders throughout Cape Town. Specific formative research has focused on stigma and discrimination, particularly through health care settings. Additionally surveillance studies have collected data on violence, sexual assault, and discrimination. Most formative research has also collected employment and salary data from participants as well.

**3. Have you planned for MSM/LGBT community capacity-building and informed participation?**

- **Secure funding to build capacity of MSM/LGBT community members**
- **Allow local groups to use resources such as meeting spaces**
- **Ensure representation of MSM/LGBT on staff**
- **Train MSM/LGBT community members to be involved as study staff to build capacity for the future.**

The GHSH CRS has invested significantly in MSM community capacity building through its current community engagement programme MSM are able to access meeting spaces in 7 communities throughout Cape Town, receive various trainings, and access funding for development of peer-led activities. Additionally, the GSH CRS staff that are responsible for MSM research are predominantly self-identified gay men. This includes the project manager, three research assistants, a study counselor, the senior outreach worker, three outreach workers, and one community intern. Extensive effort has been made to train MSM community members to be involved as study staff and to build capacity for the future. The GSH CRS has an extensive internship programme that has led to the full time employment of five MSM community members as full time staff.

**4. In dissemination results, do you have plans to work with MSM/LGBT community leaders on data dissemination and a utilization plan, including media advocacy?**

MSM community leaders, in each of the 6 communities within which the GSH CRS community programme is established, are positioned to support the dissemination of study data. Additionally, the GSH CRS outreach team also assists with data dissemination. Media advocacy beyond standard press releases has not extensively been considered previously.

**5. Do you plan to build the skills of activities to disseminate/use data locally for advocacy?**

There is substantial experience within the GSH CRS team relating to MSM advocacy. The senior research assistant completed a one-year fellowship with the AIDS Vaccine Advocacy Coalition (AVAC) in 2009 and is well positioned to translate research into community-based advocacy initiative. Additionally, current partnerships with AVAC support the GSH CRS to expand its advocacy initiatives as needed.

**Draft Crisis Communication Plan**

GSH CRS v1.0

**Crisis Communication Group**

The Crisis Communication Group will consist of a limited number of study staff members and will include:

1. PI - Dr. Linda-Gail Bekker, who will also serve as spokesperson to any media outlet or news source or key stakeholder in the event of a crisis. She is well connected with local and national government officials. She will keep lines of communication open to ensure that the Crisis Communication Group is aware of official opinion and that relevant government bodies are up-dated on study progress.
2. Project Manager - Ben Brown, who oversees community engagement and retention will be responsible for managing personnel, community communications, and operationalizing the crisis plans developed by this team. He is well connected with opinion leaders of MSM communities in Cape Town. Regular communication with key stakeholders will ensure that the Crisis Communication Group is aware of community opinion. This communication channel will also allow the Crisis Communication Group to up-date community leaders the study progress.
3. Communications Officer – Lavinia Browne - the communications officer at the DTHF, will be responsible for the development of crisis scenarios and managing communications with media.
4. Study Coordinator - Elize Batist, who has served as SCO for previous MSM studies will be responsible for coordinating participants, clinic staff, and providing updated information on the study.

These individuals have experience in handling the media, extensive knowledge of previous MSM experience in Cape Town, and the authority to make important decisions. All Crisis Communication Group members are available all hours of the day and on weekends. Their contact details are as follows:

- Linda-Gail Bekker
  - [Linda.gail-bekker@hiv-research.org.za](mailto:Linda.gail-bekker@hiv-research.org.za)
  - +27.83.266.2876
- Ben Brown
  - [Ben.Brown@hiv-research.org.za](mailto:Ben.Brown@hiv-research.org.za)
  - +27.76.687.0639
- Lavinia Browne
  - [Lavinia.Browne@hiv-research.org.za](mailto:Lavinia.Browne@hiv-research.org.za)
  - +27.72.496.9849
- Elize Batist
  - [Elizabeth.batist@hiv-research.org.za](mailto:Elizabeth.batist@hiv-research.org.za)
  - +27.82.379.9909

The GSH CRS internal communications during a crisis will be conducted via e-mail and telephone and in person. This team will have meetings as needed and will specifically convene in response to significant events. Their responsibilities will be the development and implementation of site-specific protocol for various crisis scenarios.

All members of the Crisis Communication Group will monitor the media for any news that could influence the atmosphere of good will around the study. This will include print, TV, radio, and internet media. Any significant news will be shared within the group through e-mail, telephone, and/or face-to-face meetings.

**List of steps to follow in the event of a crisis**

The crisis communications team will develop responses to individualized scenarios for the this study but will generally respond to a crisis using the following method:

1. Inform the entire communication team of potential crisis and convene either electronically or in person (preferred).
2. Gain as much information as possible regarding the situation and inform the CORE team of the crisis immediately.
3. Decide on best course of action, taking into consideration the principals of crisis management as determined by core team (transparency, early disclosure, addressing concerns, and speed).
4. Each member will then follow through with disseminating the necessary information or taking the appropriate steps for their sphere of responsibility. For example, the communications officer will work with the PI to address local media and key stakeholders, while the SCO addresses clinic staff and study participants and the project manager addresses community contacts.

**Developing Channels of Communication:**

*Internal Communications*

The members of the communications team will be contactable 24/7 with updated personal information circulated to each member. Each member will be responsible for communicating to and reporting messaging from distinct groups. For example, should someone on the clinic staff be informed of a potential crisis situation from a participant that staff member would report to the SCO who would then report to the communications team.

Additionally, in order to prepare the entire study team for a potential crisis situation, the communications team will implement a training to educate team members on what constitutes a crisis and who they should report to during such an event.

*External communications*

In the event of a crisis all staff will be instructed to respond to any media inquiry with “No comment”. All statements given to the media will be given by one of the members of the Crisis Communication Group. The Crisis Communication Group will discuss any response to the media and agree on its content before the response is made. Any live press briefings will be conducted at the GSH CRS.

An updated contact list will be developed In order to structure communications with external groups such as participants, key stakeholders, community partner groups and the media. This will be used in the event that urgent communications, messaging, or reports need to be disseminated to these contacts. The PI will be the sole spokesperson for all media and key stakeholder communications. The Project manager will be responsible for communicating directly with community contacts and partner organizations. The communications officer will be responsible for ensuring updated information regarding the study is available through all channels such as the DTHF website and social media. The SCO will be responsible for internal communications to clinic staff and participants.

**Key Messages**

Key messages will be developed prior to the launch of the study.

**References**

1. Joint United Nations Programme on HIV/AIDS. Good participatory practice. Guidelines for biomedical HIV prevention trials 2011. Geneva: UNAIDS; 2011.
2. Joint United Nations Programme on HIV/AIDS. Ethical considerations in biomedical HIV prevention trials. Geneva: UNAIDS; 2012.
3. amfAR The Foundation for AIDS Research. Respect, protect, fulfill. Best practices guidance in conducting HIV research with gay, bisexual, and other men who have sex with men (MSM) in rights-constrained environments; n.d.
4. HIV Prevention Trials Network. Ethics Guidance for Research. 2009.

Kisumu/Kenya Risk Mitigation Plan (Version 0.4)

**HPTN 075 SITE-SPECIFIC RISK MITIGATION PLAN FOR KEMRI/CDC CRS**

In collaboration with the protocol team, we have developed a site-specific risk mitigation plan, which we will implement as part of the study. The text below contains relevant measures that were already incorporated in the protocol. In addition, it contains information already provided in the site selection questionnaire. Finally, it contains issues that were covered in the provisional site-specific Risk Mitigation Plans.

**General principles**

We will implement HPTN 075 according to international guidelines for HIV prevention trials (1, 2) and for conducting research with MSM in rights-constrained environments (3). We are familiar with these guidelines and subscribe to them.

**Ongoing dynamic community consultation**

We will develop an ongoing, dynamic engagement with the MSM community and the broader general community, starting before study implementation. We will document and evaluate implementation of activities to prepare the study site as well as our efforts to optimally engage MSM and ensure that the engagement is in line with the HPTN Ethics Guidance for Research.

In addition to engaging our already established local Community Advisory Board (CAB), we have made contact with five other collaborators in Kenya to discuss HPTN 075. These include:

⮚ Liverpool Voluntary Counseling and Testing (LVCT). LVCT is an organization that is involved with providing voluntary counseling and testing services to MSM. (<http://www.lvct.org/index.php> )

⮚Keeping Alive Societies’ Hope (KASH). KASH is an organization that offers HIV intervention services to the local MSM population and has linkages with most MSM organizations as well as the most at risk populations in Kisumu County. (<http://www.kash.or.ke> )

⮚Men Against AIDS Youth Group (MAAYGO). This organization focuses on the empowerment of MSM and transgender persons to attain effective participation in society and optimal health. (<https://www.facebook.com/MaaygoMsmKisumu?filter=2> )

⮚Gay and Lesbian Coalition of Kenya (GALCK). GALCK is a national umbrella organization for gays and lesbians. (<http://galck.org> )

⮚KELIN. A national network which was established to address and respond to Legal, Ethical and human rights issues relating to health and HIV and AIDS. (<http://kelinkenya.org> ).

KEMRI/CDC also has a well-established direct collaboration with the Ministry of Health through the [Kenya National AIDS & STI Control Programme (**NASCOP**](http://nascop.or.ke/)) and we will engage them in all levels of the study development. NASCOP is also working together with GALCK, KELIN, Nyanza Reproductive Health Society (NRHS)-Kisumu and KEMRI/Welcome trust collaboration in Kilifi, Kenya. It is of note that NRHS is currently undertaking a study in Kisumu, initiated in 2012, targeting a cohort of 700 MSMs who will be followed up over 12 months with treatment offered to persons HIV infected. Our contact with NRHS will ensure that we avoid co-enrolment of participants given that we may share same recruitment area.

In general, KEMRI/CDC has expressed interest in collaborating with the above mentioned institutions in terms of exchange of technical support as well as in building the capacities of KEMRI/CDC staff and CAB in engaging the MSM population. Having shared KEMRI/CDC’s intention to be a site for HPTN 075 in Kisumu, the above mentioned organizations have indicated that they are aware of and are supportive of our being a site, including the collaborative mapping of the variety of MSM expressions in the community, development of site-specific strategies for the promotion of study awareness and acceptability in the relevant communities, and development and implementation of confidential recruitment strategies (conforming to guidelines specified in the protocol). We have also obtained a letter of support from NASCOP committing to the HPTN 075 study. We will foster ongoing consultation with these groups over the course of the study through establishing a network with organizations working with MSM populations within Kisumu; this network will be engaged though monthly meetings, consultations before any study initiation, and involvement of representatives on the Protocol Advisory Committee (PAC) and/or CAB.

In addition to our regular consultation with NASCOP, LVCT, KASH, MAAYGO, GALCK and KELIN, we will also ask MSM community representatives to identify MSM groups present in the community. If other groups are mentioned, we will contact them to establish a supportive and working relationship in terms of participant recruitment. Through ongoing involvement with these potential other groups, we will be able to identify early any potential problems related to recruitment or study participation. Furthermore, these interactions will allow us to explore whether MSM that are recruited for this study represent the MSM present in the various communities, or whether the cohort of recruited men is different in a substantive way.

One of our collaborators, MAAYGO, has agreed to be involved in the implementation of the monthly off-site peer-education visits for consenting study participants to promote retention and elicit timely feedback regarding study implementation.

**Community Advisory Board and Protocol Advisory Committee**

To facilitate ongoing community engagement, of critical importance for research with the population of MSM in sub-Sahara African settings, we will work with a CAB and a PAC. The CAB will ensure that HPTN 075 is implemented putting into consideration the local context as well as ensuring the study is implemented in an ethical, culturally appropriate and acceptable manner. The existing KEMRI/CDC CAB consists of community representatives, including government administrator (Chief), religious leaders (Christian and Muslim), health care worker (Public Health Specialist), opinion leaders, police representative, representative of MSM organizations and a human rights lawyer. The CAB will advise on general matters related to community participation. The already established PAC, will be a subsidiary of the CAB and will consist of members most familiar with MSM issues and members of the MSM community, and will assist researchers in protocol-specific matters such as education and communication materials, appropriate study materials, and may provide feedback on proposed study procedures. In case the study elicits negative reactions to the study in the general community, the PAC will consider effective responses and implement those in consultation with the emergency committee (see below) and the site Principal Investigator. The CAB is trained on research ethics and will be involved in protocol development, implementation and result dissemination. This will ensure that the community input is adequately included in all aspects of the HPTN 075 study. The PAC will play a more active role in advising on specific issues affecting the MSM populations and would play a more active oversight role on the study implementation.

Collectively with the CAB and PAC, we will identify and share concerns and priorities of the communities hosting the research, explore the study’s potential social impact, and develop a priori responses for addressing any issues.

**Emergency committee**

To facilitate a direct response to study participant- and staff-related emergencies, we will install an emergency committee of five persons, consisting of a study staff, a local government administrator, a human rights lawyer from KELIN, MSM community representative and a senior level police officer who deals with collaboration and partnership at the local police division. Through this committee, we will have the following experts available for consults if/when their expertise is needed: social worker, police official and criminal lawyer familiar with the study. The committee will also partner with the CDC Regional Security Officer (US embassy homeland security staff attached to KEMRI/CDC) who has an oversight role in handling security situations affecting US personnel and US sponsored assets. On the other hand, the KEMRI/CDC company lawyer will be contacted to facilitate legal representation of the study should the organization need legal services. The human rights lawyer in the emergency committee will facilitate the process of obtaining free legal representation for the participants through KELIN should there be a need. Additionally, this lawyer will provide local human rights and criminal law expertise/interpretation whenever requested. The emergency committee will be charged with planning approaches to any emergency situations and facilitating a response should they occur. We will establish procedures to rapidly notify this committee of any emergency situations.

**Site preparation**

KEMRI/CDC currently has a Clinical Research Center (CRC) based within the compound of Jaramogi Oginga Odinga Teaching and Referral Hospital (JOOTRH), a hospital that serves more than 10 counties in western Kenya. The CRC has been used to conduct studies on malaria, HIV and TB since the year 2000. This facility hosts a South African National Accreditation System (SANAS) accredited HIV laboratory facility, malaria Laboratory, and central pharmacy. Also within the facility is a shared clinic which houses counseling rooms, Audio Computer Assisted Self interview (ACASI) computers room (we have extensive experience in this area), clinical examination rooms and an advanced records filing room. The compound also houses conferencing facilities and a data section complete with data fax machines.

The study site is established in a manner consistent with principles of confidentiality and will take into account additional advice by the CAB and PAC. In addition, we will prepare study staff to ensure readiness for the culturally-appropriate conduct of the study and availability of culturally-competent care for all participants including men who are identified as HIV-infected at enrollment or during the study. We will ensure that the handling of all participants will be based on the ethical principles of beneficence, justice and respect of persons and in line with the HPTN Ethics Guidance for Research.

Staff who work within the KEMRI/CDC clinical research center will attend a session where they will be thoroughly sensitize and trained on measures to guard confidentiality of the participants. All study staff will receive ongoing training to interact with study participants in a non-judgmental, MSM-affirming way before the start of the study areas covered will include MSM behavior, related local laws, and challenges associated with being an MSM. LCVT will provide capacity building assistance to the study and will be consulted on technical issues related to MSM populations in Kisumu. We will ensure that all study staff are informed about the specific risks involved for study participants, study staff, and local MSM communities. Staff will engage in role play scenarios during our ongoing training where they are in the position of an MSM. Specific attention will be paid to the need for strict confidentiality. We will also provide ongoing training to research staff on the provision of referrals to counseling and social service support. We will receive support from HPTN Operations Center to accomplish this. The study staff will be required to sign confidentiality agreement forms as a sign of commitment. The confidentiality statement will explicitly indicate that disclosing any information about participants is subject to disciplinary measures up to termination. We will also build the capacities of the participating MSM organization and community groups on research ethics and aspects that would enhance community participation in the research process.

**Services**

We will offer study participants at each visit a package of clinical and standard-of-care services as part of their involvement in the study. This will include HIV risk-reduction counseling, and provision of condoms and water-based lubrication (both approved according to guidelines from the World Health Organization). These services will be provided in a manner to ensure confidentiality of the study participants. The condoms and water-based lubricants will be prepackaged in an opaque, unlabeled packaging material and will be issued to each individual participant at a private room within the clinic.

**Safety reports**

The Investigator will make safety and progress reports to the IRBs/ECs at least annually, and within three months of study termination or completion. These reports will include all unanticipated problems involving risks to human subjects or others (see also below). We will submit documentation of continuing review to the Division of AIDS (DAIDS) Protocol Registration Office, in accordance with the current DAIDS Protocol Registration Policy and Procedure Manual.

**Social harm assessment and reporting**

We will train the staff on the collection and reporting of social harms. Such events will be collected and reported by study staff according to guidelines specified by the protocol team, using a study-specific incident report form. This form will query common and MSM-specific social harms such as altered personal relationships, forced change in housing, and physical violence. The form will also include space for a written narrative to document additional details of any social harm experienced. All research staff will be trained to properly complete the form. Reports of social harms will be reviewed by the Protocol Chair, DAIDS Medical Officer, Protocol Biostatistician, the Statistical and Data Management Center Project Manager, and Leadership and the Operations Center Clinical Research Manager, quarterly or more often, if indicated, and reported to the Medical Officer together with any actions that are taken. Social harms will be summarized and reported to appropriate IRB(s) following IRB guidelines. Any emergency situations will be reported to the parties mentioned above within 24 hours.

**Study monitoring**

On-site study monitoring will be performed in accordance with DAIDS policies.

**Practical measures to mitigate risk**

In addition to the above strategies, we will implement several practical measures to mitigate risks. These include the following:

1. Identification of the study: To safeguard participants’ confidentiality, the study will not be identified as a study of MSM. Recruitment materials and messaging for the study will be focused on participation in a health and wellness study. The choice of study name will be done by the CAB and or the PAC. The CAB and the PAC will be sensitized on the intentions of the HPTN 075 study then would be guided by the staff as well as the local MSM organizations who have agreed to collaborate with us, to choose a name that is culturally appropriate and friendly toward the MSM health and confidentiality needs.

2. Engagement of staff at Ministry of Justice, National Cohesion and Constitutional Affairs, the Ministry of Interior and Coordination, Ministry of Welfare and Social Services and the Department of Public Prosecutions: To ensure that we have the maximum amount of awareness of and support for this project, we will meet with the staff associated with the above country agencies. We will schedule an appointment before community engagement begins to present key aspects of the study. In addition, we will review our plans for response to interference (see below) and ask for comments. If needed, our plan will be amended. The staff will be invited to visit our site if/when they are in Kisumu. We will email or call the staff monthly to keep them abreast of the study progress and any issues. In case a legal issue arises, we will call on these key staff and ask for assistance. We will also develop a memorandum of understanding outlining how they would be engaged as partners in the research implementation process. The memorandum is anticipated to enlist their support to the study implementation. At study enrollment, participants will be given written information about the availability of social services available outside the study that would be expert in dealing with issues relevant to MSM (e.g. Liverpool VCT, Ministry of Welfare and Social Services satellite offices at the Jaramogi Oginga Odinga Teaching and Referral Hospital (JOOTRH) which is situated adjacent to our site). Counseling services at Liverpool VCT are available 6 days per week and are available at JOOTRH 5 days per week. These local services will be important in the event that a participant withdraws from the study and loses access to study counselors.

3. Community engagement strategy: We will work together with NASCOP and existing MSM and health promotion organizations in educating and sensitizing the community specifically on the health aspect of the MSM population by highlighting that research will help understand better how MSM population contribute to the high HIV incidences in the community and the need to have an HIV management strategy that targets the MSM. We will build the capacities of representatives of MSM and health promotion organization so that they own the process with technical input from the study staff. This may help delink the study from possible MSM advocacy allegations that could place the organization’s premises and staff at risk especially when operating in an MSM hostile community. We will include in our training plans, representatives from the Ministry of Justice, National Cohesion and Constitutional Affairs, the Ministry of Interior and Coordination and the Department of Public Prosecutions. The involvement of these government organs will ensure public confidence in the study objectives and may provide a platform for effectively resolving any legal issues that may arise during the study implementation process as well as ensure their support of the study in achieving its research objectives.

4. Screening strategy: The screening questionnaire will ask men about their sexual behavior with male and female sexual partners in the past 3 months in addition to several other questions. Potential participants will not be asked to self-identify their sexual orientation. In this way, MSM can be selected for participation through the screening questions.

5. Study participation: The clinic section at the KEMRI/CDC CRC is shared among different studies which currently include HIV and TB studies. The center was also previously used as a clinic for malaria studies. The clinic has been designed in such a way that study participants from multiple studies share the same rooms and in some cases staff. Therefore, it would be very difficult for a non-participant to know the type of study each participant is coming to attend. Only the staff within the clinic rooms are able to determine this information. From previous experience with other Most at Risk Persons (MARPS) studies, the privacy of our participants has been effectively handled.

6. Data management: Detailed locator information, collected at the study Screening Visit and actively reviewed and updated at each subsequent visit, will be stored separately from other study data and kept in double locked cabinets. For each study participant, a unique ID will be generated and assigned; there will be no personal identifiers. Only specific study personnel will have access to any hard copies of data and the computer files.

7. Monitoring staff interactions with participants: We intend to have an exit questionnaire at the end of every visit to capture any areas the participants are not satisfied with. The questionnaire will be administered using ACASI in order to ensure privacy and objectivity in the participant responses. The responses will be analyzed in real time. Any issues identified that may have negative effect on the participation will be responded to in real time.

8. Staff’s maintenance of confidentiality: Staff will sign confidentiality statements at recruitment and on annual basis regarding sharing any participant information, ensuring the privacy of participants. The confidentiality statement will give examples of the kinds of information that should be kept confidential and will mention that disclosing any information about participants is subject to disciplinary measures up to termination according to the KEMRI /CDC policy. Should there be allegations of staff breaching the confidentiality of the any participant, thorough investigations will be done through the KEMRI/CDC human resource department and appropriate disciplinary action will then be implements according to KEMRI/CDC disciplinary policy upon conviction.

9. Sensitivity training of CAB: The KEMRI/CDC CAB is well established and as mentioned earlier, comprises of a diverse group of persons from Kisumu. Importantly, an MSM community representative and a human rights lawyer are members of this CAB. The CAB is in the process of recruiting a representative from the police service. Due to the sensitive nature of this study, we will make clear to potential and current CAB members that participation in the CAB might pose a risk for members.

The CAB has already been trained on research ethics and basics on HIV. They have long term experience in representing the views of the most at risk groups. Our organization is in the process of contracting LVCT to conduct a comprehensive training on MARPs with specific emphasis on MSMs and commercial sex workers. LVCT center has a comprehensive training curriculum that they have used in training other partners who work with MSMs.

The CAB members will therefore sign a memorandum of understanding with the study regarding their role in maintaining confidentiality and will be encouraged to include in their terms of reference, measures they would take as a CAB in handling situations where one of their members breaches confidentiality, emphasizing on the legal implications of such action.

The CAB leaders will also outline, with input from the CAB members, the key activities of the CAB with regard to their role and purpose. They will then focus on the development of procedures to guide the logistical operation of the CAB, the development of guiding principles that will provide the basis for community trust and representation, and the establishment of leadership and decision-making protocols. These functions will be formalized in writing and monitored by a rotating team of persons dedicated to evaluation of the effectiveness of the CAB. The evaluation will occur quarterly and be shared with the study staff and community.

10. Complaints about treatment by staff: There will be a multipronged approach that will be used to address complaints about the staff. The choice of each approach will be based on whether it affects the individual participant only or the entire group of participants and whether the complainant would like to communicate anonymously or would be comfortable engaging the supervising staff directly.

*Individual anonymous complaints*

We plan to have a suggestion box placed at the outside of the premise so that participants would anonymously place their written suggestion in the box. The box contents will be reviewed weekly. The study will also identify a CAB and or PAC representative whom participants would be encouraged to contact whenever they have issues. The representative(s) will in turn relay the information to the study supervisors and provide feedback to the participants concerned.

*Individual direct complaints*

We will have study hotlines with the hotline numbers shared to all participants. They will be encouraged to contact the study supervisors through these hotlines and the complaints will be documented by the persons receiving the calls and the documentation reviewed by the supervisors for action. The participants will also be encouraged to walk into the clinic and seek audience with the PI, study coordinator or a designated staff to further discuss the issue and decide on a mutually agreeable resolution. Should they feel that their grievances are not adequately addressed they would be encouraged to contact individuals and institutions as listed in their consent documents for further redress.

*Group complaints*

The study will create forums for group complaints whereby participants will be asked to voluntarily participate in the forums. During these sessions the participants will voluntarily forward their complaints after which amicable solutions to the problems would be found and implemented and periodic feedback sought.

11. Response to outside interference: The constitution of Kenya under the bill of rights Article 27 outlaws discrimination against individuals based on tribe ethnicity or race but does not specifically include discrimination based on sexual orientation. However human rights organizations argue that this bill cuts across all citizenry and that includes MSMs. The same bill of rights emphasizes that all citizens have the right to health services (This would be the key constitutional basis of KEMRI/CDC’s engagement with the MSM populations).

On the other hand, sex acts between men are illegal under Kenyan statutes and carry a maximum penalty of 14 years' imprisonment. The penal code section 162 of the laws of Kenya, categorized under unnatural offences, criminalizes homosexuality. It states that any man caught having sex with another man is liable to imprisonment of up to 14 years. Any coercive sex between men attracts a maximum of 21 years in prison and an attempt to commit sexual act among males or indecent sexual acts between males in public or in private attracts 5 years jail term.

Based on the principle of the right to health for all citizens, and considering that MSM and MARPs contribute to one third of the HIV infections in Kenya, the Ministry of Health developed a national guideline for sex workers in 2010. The guideline recognized MSMs as one of the MARPs groups that should be targeted by special HIV interventions in the effort to reduce the HIV incidence and prevalence in Kenya. Therefore, in this perspective, institutions that provide health and wellness services to MSMs are basically recognized by the Ministry of Health and hence guaranteed government support for such projects. Therefore, the likelihood of law enforcement and criminal justice system interfering with the HPTN 075 study is very minimal. However, in an unlikely event that there is such interference, KEMRI/CDC will engage the Ministry of Health (NASCOP) and collaborating human rights organizations (e.g. KELIN) for technical support and legal representation. KEMRI/CDC also has a company lawyer who represents the organization whenever a need arises. The study staff will ensure that there is a close relationship with the local police service and also involve the government administrators by sensitizing them on the HPTN 075 activities. Currently the police service has a section that collaborates with health research and health service providers for MSM and CSW populations in Kisumu. HPTN 075 Kisumu site will obtain written permission and guarantee from Police, local administration and the Ministry of health at county level that will ensure no interference in the activities related to the HPTN 075 study. The government administrators will be provided with regular updates regarding the progress of the study and will be involved and consulted on issues touching on the security of our staff and participants whenever the need arises.

In the event of interference from hostile community members, the emergency committee will be notified and the CDC Regional Security Office (CDC RSO) will also be notified. A remedial action will then be planned by the committee with the advice from the CDC RSO. Contacts will be made with the Kenya government ministries in charge of security and legal issues, to alert them of the incident. Where possible, a meeting could be arranged with their representatives to develop a common plan of action. The plan will then be implemented in partnership with the government ministries while ensuring they take a leading role in resolving the incident when possible.

If KEMRI/CDC is chosen as a site for HPTN 075, as part of the site activation process, the above plan will be verified by a representative of an organization outside of KEMRI/CDC. The verification will include, for instance, that written permission of cooperation and support of HPTN 075 from police, local administration, and the MOH at the country level has been obtained.   This will be included in the SSP.

KEMRI/CDC has had to effectively deal with outside interference in the past. One example is when the offices of one of our key collaborators in MSM research was attacked by community members and their staff arrested by the police. The organization was accused of promoting MSM activities in the area. The organization had been contracted by NASCOP to conduct a survey on HIV in the area and in the process was providing reimbursement for participation in the survey, an act that was erroneously seen by the community members as providing incentives to the boys to join MSM activities. The KEMRI/CDC staff, the CDC RSO together with a human rights lawyer and representative of the MSM umbrella organization intervened by talking to the police to explain that the group engages in HIV/AIDS prevention work. Our staff then talked to the community at barazas on an ongoing basis to educate people regarding the fact that many MSM are married and thus by protecting MSM health we are protecting the general community health.

A second example is when KIPE was raided by persons in the Muslim community. The community members believed that the group was recruiting young people to become MSM and demanded the site be closed. We both educated the community and also discussed the possibility of relocating the offices to avoid future similar problems. It was decided to move the offices to a more secure area that was less residential and thus had fewer children in the area. They are now located in central business district.

In order to be fully prepared to respond effectively to an attack or raid associated with the HPTN 075 study, we will work with the protocol team to develop plans to respond to various scenarios. We will then identify roles for each of our team members and practice the actions in the plan before the onset of the study and at least 2 times per year.

Communications with the media will be handled by the KEMRI/CDC communications department and/or the local MSM collaborative network. We have also developed a draft communications document (Appendix 1) with key information regarding the study that will be given to persons in case of occurrence of any such incidents as those noted above. This document will ensure that all parties have the same information to offer if contacted by media or others.

The above mentioned resolution mechanism will also be included in a Site Specific Procedure (SSP) guideline that will be shared with CDC RSO, PAC, CAB, and Emergency Committee. The IRBs will be notified promptly within 3 reporting days of any emergency situations from the community or authorities that may affect the study implementation.

This Risk Mitigation Plan will be revisited at least annually. It will be revisited based on engagement with community, IRBs, authorities in order to maximize the rights, interests and welfare of the participants. Similarly, the plan will be revised in the face of any unexpected policy changes, etc. that may occur in the duration of the study.

We have also listed in appendix II a list of questions that highlights our sight preparedness.

**References**

1. AmfAR The Foundation for AIDS Research. Respect, protect, fulfill. Best practices guidance in conducting HIV research with gay, bisexual, and other men who have sex with men (MSM) in rights-constrained environments; n.d.
2. Joint United Nations Programme on HIV/AIDS. Good participatory practice. Guidelines for biomedical HIV prevention trials 2011. Geneva: UNAIDS; 2011.
3. Joint United Nations Programme on HIV/AIDS. Ethical considerations in biomedical HIV prevention trials. Geneva: UNAIDS; 2012.
4. Ministry of Public Health and Sanitation (Kenya). National guidelines for HIV/STI programs for sex workers: NASCOP: 2010
5. Rennie S, Sugarman J & HPTN Ethics working group. HIV prevention trials network ethics guidance for research. NIAID; 2009. <http://www.hptn.org/web%20documents/EWG/HPTNEthicsGuidanceV10Jun2009.pdf>
6. Newman, SD, Andrews, JO, Magwood, GS. et al. Community Advisory Boards in Community-Based Participatory Research: A Synthesis of Best Processes. Preventing Chronic Disease: Public Health Research, Practice, Policy 2011; 8. <http://www.cdc.gov/pcd/issues/2011/may/10_0045.htm>

**Appendix 1- Draft communications document for HPTN 075**


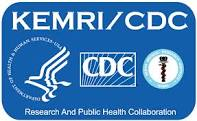


⮚HPTN 075 is an observational cohort study being carried out by KEMRI/CDC.

⮚The study will determine the feasibility of recruiting and retaining roughly 400 men who have sex with men (MSM) in Kisumu and at three other sites in sub-Saharan Africa in preparation for future HPTN studies in the region.

⮚The study aims to help MSM avoid HIV infection, improve their overall health and help them cope with negative social stigma.


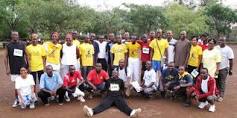
*This study is important to the Kisumu and surrounding community because many MSM say that they maintain concurrent relationships with men and women. Thus by improving the health of MSM, we are improving the health of the community.*

APPENDIX II

Questions for Researchers to Ask for MSM/HIV Research

| **RESPECT** | Status | Notes |
| --- | --- | --- |
| Have you included the MSM/LGBT community in:  Engagement rules | Yes | KEMRI/CDC have progressively engaged the MSM community in past studies i.e. Formative research on recruitment strategies for high risk populations. We have a good a working relationship with MSM in Kisumu and have been providing HIV prevention information and referral for HIV testing services to our other partners (LVCT Care and Treatment, Nyanza Reproductive Health Society (NRHS), KIPE and MAAYGO’s outreach services since 2009. We also have included an MSM in our CAB. |
| Situational assessment | Yes | We have close working relationship with NHRS who works with MSM populations in Kisumu. |
| Have you assessed the relevance of the research and potential reactions from greater community structures? | Yes | We have assessed the relevance of the research and reactions from the community. The National AIDS Control Council (NACC) of Kenya has been at the forefront of efforts to document the existence of MSM and their vulnerability to HIV in the face of denial and rhetoric. NACC, through the Kenya National AIDS Strategic Plan (KNASP) and in conjunction with partners, developed a strategy for engaging MSM in the national response to HIV. We will benefit from this plan in that it works to increase the number of organizations delivering services to MSM. Our CAB has a diverse membership and will further reach out to assess the relevance of the research and community response within 2 to 3 months of our site approval. In case of any negative reactions, the solidarity in engagement with other partners will play a greater role in their resolution. |
| Have you assessed the interest amongst the MSM/LGBT community, as well as current infrastructure (or lack thereof)? | Yes | Our in press publication, “MSM in Kisumu, Kenya; Comfort in accessing health services and willingness to participate in HIV prevention studies”, provides evidence that the vast majority (49/51; 96.08%) of MSM are willing to be contacted to participate in HIV research studies. The formal and informal MSM groups have shown interest in health research and are currently developing an advisory committee to help MSM to discern their participation in such activities. We plan to work closely with this committee. |
| Have you assessed the willingness of your research institution to Respect, Protect, and Fulfill rights of participants? | Yes | With the support accorded to previous studies involving MSM, we are certain and have confirmed commitment of KEMRI/CDC |
| Have you developed an MOU with community-based organizations—clearly involving them in all aspects of the research? | To be Done | We plan to have an MOU with all potential partners mentioned in this document working with minority groups including MSMs within 2-5 months of our site notification of approval. They all are supportive and are willing to work with us in protecting participants’ rights during the study. |
| Have you clearly defined roles and responsibilities of all stakeholders? | To be Done | We have not developed a stake holder directory for this study, however, we plan to do this 2-5 months following approval notification and prior to study initiation. |
| Have you conducted a comprehensive identification process with stakeholders including:  Community stakeholders, NGOs, CBOs, community groups, informal networks, etc. | To be worked on further | As a research organization, we have existing structures in collaboration with other stakeholders. We will conduct a full scale identification process with stakeholders upon official notification of protocol approval within 2-5 months of study approval. |
| Government ministries, leaders, etc. | To be worked on further | As a research organization, we have existing structures in collaboration with other stakeholders. We will conduct a full scale identification process with stakeholders within 2-5 months of our site notification of approval. |
| Local health care facilities and services | To be worked on further | As a research organization, we have existing structures in collaboration with other stakeholders. We will conduct a full scale identification process with stakeholders within 2-5 months of our site notification of approval. |
| Local religious leaders | To be worked on further | As a research organization, we have existing structures in collaboration with other stakeholders. We will conduct a full scale identification process with stakeholders within 2-5 months of our site notification of approval. |
| Media | To be worked on further | As a research organization, we have an established communications department and structures in collaboration with other stakeholders. The KEMRI CGHR communications team will work hand in hand with the key study staff in their interaction with the media and in practicing individualized scenarios for this study. We will conduct a full scale identification process with stakeholders within 2-5 months of our site notification of approval. |
| Have you engaged government, while first discussing effective models of engagement with community representatives? | To be worked on further | Government engagement has occurred in past and ongoing studies at KEMRI CGHR site through representatives of Kenyan Ministry of Health as well as provincial administration authorities. This will be revisited following approval and throughout the course of the study |
| Have you secured funds for community involvement (e.g., providing financial Incentives, etc.)? | No | Funding for community involvement occurs through a variety of programmes conducted at the KEMRI CGHR site. All research projects budget for community engagement activities. Financial incentives are not used for standard community engagement activities, however, should participants incur transport costs, they are reimbursed. |
| Will you start by conducting formative research activities to learn more about the target populations and their priorities? (This would also include learning about what prior research has been conducted in this population and what are the local perceptions of this research [both from MSM and from non-MSM].) | Yes | We would conduct formative research to learn more about MSM in the area and their priorities. Howeever, it is of note that we have already conducted formative research on MSM in the area on the following topics: new technology use for study participants’ identification through biometrics, stigma, discrimination, access to healthcare services, membership in support groups and recruitment strategies. The papers resulting from this research can be found in the reference section. |
| Have you included research on human rights protections/violations within the research context? | To be worked on further | Human rights protections and violations have been explored in the ongoing HPTN 052 and in the high risk cohort studies in 2009 through to 2012. We are considering undertaking a study in the aspect of human rights protections and violations in the formative research at the beginning of study. |
| Will you provide research literacy training to key stakeholders?  Local NGOs, CBOs, informal networks of MSM/LGBT | To be worked on further | Research literacy training has been provided to stakeholders and participants in the KEMRI CGHR through outreach programmes in the form of workshops, and discussion groups specifically focusing on HIV prevention research.  This will form part of our pre study initiation engagement and sensitization activities within 2-5 months following site notification of approval. |
| Healthcare service providers | To be worked on further | This will form part of our pre study initiation engagement and sensitization activities within 2-5 months following site notification of approval. |
| Media | To be worked on further | This will form part of our pre study initiation engagement and sensitization activities within 2-5 months following site notification of approval. |
| Government | To be worked on further | This will form part of our pre study initiation engagement and sensitization activities within 2-5 months following site notification of approval. |
| Influential community leaders | To be worked on further | This will form part of our pre study initiation engagement and sensitization activities within 2-5 months following site notification of approval. |
| **PROTECT** | Status | Notes |
| Have you developed policies for dealing with hostile/intrusive media, media that may blame MSM for ‘spreading HIV’ in a country? | To be worked on further | KEMRI/CDC has an established communications department with SOP for interaction with the media. This policy stipulates specific procedures for clearance and approval of expert authorities. It also includes staff training for media interaction. Within 2-5 months following site notification of approval, we will have in place policies for dealing with hostile/intrusive media that may blame MSM for spreading HIV in Kenya. |
| Have you developed certificates of confidentiality to help participants feel safe, knowing that their information will not be shared with others? | To be done | There is a confidentiality policy included in the KEMRI/CDC employment contract letter. We shall develop a study specific confidentiality certificate and staff members will be required to sign this agreement prior to working with participants or participants’ data. Participants will be informed of this practice and be able to see the confidentiality agreements should they wish to. This certification will be done within 2-5 months following site notification of approval.  . |
| Have you developed personal identifiers that protect people’s identities, or considered conducting research anonymously? | To be done | In all our previous studies, each participant have been assigned an identification number relating to all study specific laboratory specimen, reports, study data collected, processes, and administrative forms. We will develop personal identifiers for the HPTN 075 study within 2-5 months following site notification of approval. |
| Have you ensured safe storage of any data that would link participants’ sexual orientation information or behavioral practices? | Yes | All our local databases are secured with password-protected/access regulated systems. Forms, lists, logbooks, appointment books, and any other listings that link participant ID numbers to other identifying information are and will be stored in a separate area with limited access and regulated by a records technologist. A participant’s study information will not be released without the written permission of the participant, except as necessary to authorized medical care providers and for monitoring by the representatives of the HPTN 075, regulatory authorities, and/or the site IRB/EC. |
| **FULFILL** | Status | Notes |
| Have you (or others) conducted formative research activities to learn about:  MSM needs and specific priorities | To be explored on further | Our past study on MSM in Kisumu (2009) partly sought to identify specific needs. It is likely, however, that more research is needed as the needs are likely to change over time. We intend to do this formative research within 2-5 months following site notification of approval. |
| Prior research in this community | Yes |  |
| Local perceptions of past research (both from MSM and from non-MSM) | No |  |
| Have you (or others) conducted formative research to learn more about and address structural drivers of HIV and STI risk when researching MSM in low- and middle-income countries? | No | We are planning to address the aspect of structural drivers of HIV and STI risk as well as human rights protections and violations in the formative research that will be initiated within 2-5 months following site notification of approval. |
| Criminalization | No | We intend to include this as part of the formative research noted above. |
| Stigma and discrimination | Yes | This was addressed in our MSM study regarding comfort in accessing health services and willingness to participate in research studies. |
| Violence/sexual violence | No | We intend to include this as part of the formative research noted above. |
| Poverty | No | We intend to include this as part of the formative research noted above. |
| Have you planned for MSM/LGBT community capacity-building and informed participation? | No | The KEMRI CGHR community capacity building has been done through its current community engagement programme. The MSM groups currently access meeting spaces at our site and would be easily accessible for onward trainings and engagement in the context of HPTN 075.  Funding for capacity building of staff and community has come from specific KEMRI/CDC funded research projects. We will identify and engage MSM other than those from groups with whom we already have a realtionship.  We will conduct these trainings within the 2-5 months period following approval and refresh as appropriate during the course of the study. |
| Secure funding to build capacity of MSM/LGBT community members | No |  |
|  |  |  |
| Ensure representation of MSM/LGBT on staff | No |  |
| Train MSM/LGBT community members to be involved as study staff to build capacity for the future | No |  |
| Do you plan to build the skills of activists to disseminate/use data locally for advocacy? | To be done |  |
| Allow local groups to use resources such as meeting spaces | Yes | We have many times provided space for the local MSM group meetings at our site. |
| In disseminating results, do you have plans to work with MSM/LGBT community leaders on data dissemination and a utilization plan, including media advocacy? | Yes | In a past formative research study involving the MSM community (March - September 2009), we involved the MSM in dissemination of the study results. |

Soweto/South Africa Risk Mitigation Plan (Version 0.4)

**HPTN 075 SITE-SPECIFIC RISK MITIGATION PLAN FOR THE HPTN CRS of THE SOWETO CTU AT THE PERINATAL HIV RESEARCH UNIT (PHRU)**

**Protocol Title:** CHARACTERIZING THE FEASIBILITY OF HIV PREVENTION COHORT STUDIES AMONG MEN WHO HAVE SEX WITH MEN (MSM) IN SUB-SAHARAN AFRICA

**Study design:**

As outlined in Section 2.3 of the protocol, this is an observational cohort study of approximately 400 MSM - about 100 men at up to four clinical research sites (CRSs) in up to four countries in SSA. The cohort will be accrued over a 6-month period, with no replacement for participants lost during the 52 weeks (12 months) of follow-up. After Screening, there will be five study visits (including enrollment and quarterly follow-up visits). Each visit will include physical examinations, collection of biological samples, HIV testing, HIV risk reduction counseling, behavioral assessments, and assessment of the social impact related to the study participation (see Schedule of Study Visits and Procedures, Appendices I-III). A subset of visits will include STI testing. The study will be implemented according to international guidelines for HIV prevention trials and for conducting research with MSM in rights-constrained environments. (3, 4).

In collaboration with the protocol team, we have developed a site-specific risk mitigation plan, which we will implement as part of the study. The text below contains relevant measures that were already incorporated in the protocol. In addition, it contains information already provided in the site selection questionnaire.

**General principles**

The HPTN 075 study will be conducted at the HPTN CRS of the Soweto CTU at the Perinatal HIV Research Unit. (PHRU) We will implement HPTN 075 according to international guidelines for HIV prevention trials (1, 2) and for conducting research with MSM in rights-constrained environments (3). We are familiar with these guidelines and subscribe to them.

The legal position of MSM in South Africa is protected by the constitution of South Africa (as embodied in the Bill of rights) where discrimination based on sexual orientation is prohibited.

Same-sex marriage has been legal in [South Africa](http://en.wikipedia.org/wiki/South_Africa) since November 2006 once the [Civil Union Act](http://en.wikipedia.org/wiki/Civil_Union_Act,_2006) came into force.

The South African National AIDS Council (SANAC), a voluntary association of institutions was established by the national cabinet of the South African Government to build consensus across government, civil society and all other stakeholders to drive an enhanced country response to the scourges of HIV, TB and STIs.

SANAC has 17 very diverse civil society sectors and these represent specific sections of society.

MSM is specified as a Key Population in the South African National Strategic Plan 2011-2016. It is situated in a broader LGBTI group. As recognition and acknowledgement of the organisations and service providers who have done most of the groundwork in research, advocacy, training,

treatment, support and care for the LGBTI community, these service providers were invited to participate in SANAC.

The Anova Health Institute with whom the HPTN CRS at the Soweto will be collaborating to conduct this study has representation on SANAC, as a founding member of the LGBTI sector. . Anova/Health4Men’s training and advocacy manager is also the Vice Chair of SANAC. It is worth noting that Anova Health’s Simon Nkoli MSM clinic was based at the PHRU for a few years before relocating into Soweto.

**Ongoing dynamic community consultation**

We will develop an ongoing, dynamic engagement with the MSM community and the broader general community, starting before study implementation. We will document and evaluate implementation of activities to prepare the study site as well as our efforts to optimally engage MSM. Community engagement is a cornerstone of the guidance points of the HPTN Ethics Guidance for Research document. (Revised June 10, 2009) (4)

We will collaborate with the Anova Health Institute’s Health 4Men (H4M) services to conduct HPTN 075. Health4men has been active in this community for a number of years and has good relationships with many stakeholders in the Soweto community. MSM HIV services and prevention activities are part of the South African National Strategic Plan for HIV and Anova’s Health4men program is a lead partner with government services across the country to do this. Anova Health is also a member of the South African National AIDS Council’s LGBTI sector, which is the recognized community forum.

We’ve had face-to-face meetings with H4M, and their community teams. This group has indicated that they are aware of and are supportive of the conduct of the study, including the collaborative mapping of the variety of MSM expressions in the community, development of site-specific strategies for the promotion of study awareness and acceptability in the relevant communities, and development and implementation of confidential recruitment strategies (conforming to guidelines specified in the protocol). We will foster ongoing consultation with this and other relevant groups over the course of the study through the involvement of representatives on the CAB, regular CAB meetings and periodic consultations with the relevant stakeholders dependent on the prevailing issues. Strategies for the promotion of study awareness and acceptability in the community, and for recruitment will be developed in consultation with the community. Peer outreach, participant referral by friends, indirect recruitment and the use of key figures and trusted persons with access to MSM networks are recognised methods to recruit and encourage MSM to participate. (Protocol Section 3.3 and 7.0)

Retention will be promoted by building trust in the study through the involvement of the study community at large before the implementation of the study

We expect that this involvement with the community will contribute to the community’s trust in the study and promote study retention. As part of the development of the recruitment strategies, we will ask community representatives to further identify the various MSM groups present in the community. We will use their projections to collaboratively review recruitment outcomes. Through ongoing involvement with these groups, we will be able to identify early, any potential problems related to recruitment or study participation. Furthermore, these interactions will allow us to explore whether MSM that are recruited for this study represent the MSM present in the various communities, or whether the cohort of recruited men is different in substantive way.

Health4Men’s community outreach workers (known as “ambassadors”) will also support the implementation of the monthly off-site peer-education visits for consenting study participants to promote retention and elicit timely feedback regarding study implementation.

**Community Advisory Board and Protocol Advisory Committee**

To facilitate ongoing community engagement of critical importance for research with the population of MSM in sub-Sahara African settings, we will work with our Community Advisory Board (CAB) and a Protocol Advisory Committee (PAC). Establishment of a PAC is mandated by the protocol. (see section 2.3)

Existing CABs at the Soweto CTU are being expanded to include adequate MSM representation. Consultation and collaboration between the Soweto CTU and H4M has already begun to address this. The PHRU CAB already consists of community representatives, religious leaders, health care workers and opinion leaders; representation of MSM organizations is being fast tracked presently; If possible, a human rights lawyer will advise on general matters. Whilst the existing CABs at the Soweto CTU have not experienced any problems in the past, the site acknowledges that the expansion of the CAB to include MSM representatives could potentially pose a risk to all the members and the MSM representatives specifically. The CAB will be engaged in how this should be managed and a process developed to manage any negative situations. The PHRU Prevention CAB currently has as its chair, a representative from the South African Police Services (SAPS). A social worker working at the SAPS is also on the PHRU Prevention CAB.

CAB activities are currently overseen and facilitated by the Soweto CTU CAB coordinator. Meetings are convened on a monthly basis and proceedings minuted. CRS Investigators or study coordinators often participate in CAB meetings in order to discuss new protocols or provide updates on studies in progress.

Per protocol, ‘implementation of the study will be preceded by site preparation and community consultation to ensure readiness for the culturally-appropriate conduct of the study and availability of culturally-competent care for men who are identified as HIV-infected at enrollment or during the study.’

A PAC will be established in consultation with the CAB and the Protocol team. It is envisaged that the PAC will consist of members most familiar with MSM issues and members of the MSM community, and assist researchers in protocol-specific matters such as education and communication materials, appropriate study materials, and may provide feedback on proposed study procedures. In case the study elicits negative reactions to the study in the general community, the PAC will consider effective responses and implement those in consultation with the emergency committee (see below) and the site Principal Investigators.

Collectively with the CAB and PAC, we will identify and share concerns and priorities of the communities hosting the research, explore the study’s potential social impact, and develop a priori responses for addressing any issues.

**Emergency committee**

To facilitate a direct response to study participant- and staff-related emergencies, we will install an emergency committee of at least five persons, consisting of study staff and community representation. The Soweto CTU is on the campus of the Chris Hani Baragwanath Academic Hospital; and already benefits from its on-site security. Additional security will be arranged should the need be identified.

The committee will be charged with planning approaches to any emergencies and facilitating a response should they occur. We will establish procedures to rapidly notify this committee of any emergencies. Evacuation plans tailored for this clinic will added to the existing safety and evacuation plans for the CTU. The plans will be distributed or made available to all facility staff to assure prompt and efficient implementation. Site-preparation and study initiation will include dealing with potential emergencies. Participant information leaflets will include the Principal Investigator contact details in order that participants have an emergency contact number. Strategically placed noticeboards (as for the general emergencies) will be placed in the clinic with the names and telephone numbers of clinic staff, emergency medical personnel, and emergency service systems.

In the manner that the PHRU already conducts training for emergency situations and fire drills, potential scenarios (and management plans) that this clinic might face will be discussed as well as role played by the study staff and relevant staff noted above in realistic and frequent practice mock emergency or disaster drills.

It must be noted that this research will take place in an environment with no legal restrictions on MSM, and where there has been a history of an MSM focused clinic, and referral from Health4Men’s MSM specific health services in Soweto.

The emergency committee at the PHRU is yet to be established but will at a minimum consist of the following:

- R Panchia – Site PI
- Ms N Barnabas – PHRU CAB Coordinator
- Mr J Cindi – PHRU clinic coordinator
- Mr R Naidoo – PHRU Operations manager
- Mr G De Swardt – Health4Men Program Director
- Social worker – to be named
- A legal representative of the Wits Health Consortium to advise on criminal and human rights matters. In addition, llegal assistance and advice will be sought from SECTION27, a public interest law centre that seeks to influence, develop and use the law to protect, promote and advance human rights.

This committee will engage with the representatives of the local police station (Diepkloof Police station, Soweto in this case) in order to make them aware of the study as well as the site’s concerns of the potential for harm for the MSM population in general and then more specifically for participants of this study.

An SOP will govern the functioning of the committee. The IEC/IRB will be notified of and emergent situation within three days of occurrence of the event.

**Site-preparation**

The study site will be established in a manner consistent with principles of confidentiality and taking into account advice by the CAB and PAB. In addition, we will prepare study staff to ensure readiness for the culturally appropriate conduct of the study and availability of culturally competent care for all participants including men who are identified as HIV-infected at enrollment or during the study. In addressing the needs of such key populations, we will undertake sensitisation training amongst clinical staff involved in studies, which include members of key populations such as sex workers, and MSM. As such, our staff will be fully equipped and aware of the concerns and contextual dynamics of MSM. Further to this, we have a culture of good clinical practice (GCP), with all study staff being GCP certified. In line with all study preparation, we will ensure that all study staff are aware of and equipped to disseminate information about the specific risks involved for study participants, study staff, and local MSM communities. All study staff will receive study specific training to interact with study participants in a non-judgmental, MSM-affirming way before the start of the study. The HPTN CRS at the PHRU will ensure that all study staff are informed about the specific risks involved for study participants, study staff, and local MSM communities. Areas covered during the training will include legislative frameworks affecting MSM, community wide challenges and individual MSM risks and behaviors.

Health4Men has a well-established national training curriculum for this, and is currently working to roll this out in primary health facilities in the area. The study site staff will receive the same level of “MSM Competency” training. All training that the staff receive will be followed up with mentoring for the general staff and clinical staff i.e. doctors and nurses. This will be part of the ongoing support provided by H4M team. Initial and ongoing training and mentorship may involve the use of case studies and role-plays as part of the training. This will enable staff to discuss difficult situations, while understanding how they can tactfully address any sensitive issues. Attention will be given to the need for strict confidentiality. As a part of study training, we will also train research staff on the provision of referrals to counseling and social service support. We will receive support from HPTN Operations Center to accomplish this.

**Services**

As with other Wellness programmes conducted by PHRU and H4M, we will, offer study participants at each visit a package of clinical and standard-of-care services as part of their involvement in the study. This will include HIV risk-reduction counseling, and provision of condoms and water-based lubrication (both approved according to guidelines from the World Health Organization) as well as treatment or referral for any clinical conditions noted during visits. All services will be provided in a manner to ensure confidentiality of the study participants.

Health4men have been working closely with the Department of Health tertiary hospitals in and around Gauteng province to strengthen relationships with the specialists in the hospital setting, have trained, and mentored them at various hospitals on MSM specific issues. H4M will continue to strengthen these relationships and Dr Radebe, the H4M clinical operations manager holds regular clinical forums /meetings with the specialists at all hospital to support this initiative. This will support any necessary referrals from the study. H4M also have representation on the LGBTI sector committee of the South African National AIDS Council (SANAC) that advocates for availability of services for key population individuals in the public health sector without stigma and discrimination.

H4M educational materials in leaflet form on a range of relevant health related issues (for example, STIs in MSM, HIV prevention, and antiretrovirals and side effects) will be available on site for distribution to participants. Participants will also be made aware of the “H4M Connect” mobile phone based MSM service, which provides information and a question and answer facility on HIV and sexual health.

**Safety reports**

Clinical events and social harms will be reported to the IRB/EC per protocol (Section 5.0) and the local regulatory requirements.

The Investigator will make safety and progress reports to the IRBs/ECs bi-annually per the local IRB/IEC requirement, protocol team directive, and protocol and within three months of study termination or completion. These reports will include all unanticipated problems involving risks to human subjects or others (see also below).

We will submit documentation of continuing review to the Division of AIDS (DAIDS) Protocol Registration Office, in accordance with the current DAIDS Protocol Registration Policy and Procedure Manual.

**Social harm assessment and reporting**

We will train the staff in the collection and reporting of social harms. Such events will be collected and reported by study staff according to guidelines specified by the protocol team, using a study-specific incident report form. This form will query common and MSM-specific social harms such as altered personal relationships, forced change in housing, and physical violence. The form will also include space for a written narrative to document additional details of any social harm experienced. All research staff will be trained to properly complete the form. Reports of social harms will be reviewed by the Protocol Chair, DAIDS Medical Officer, Protocol Biostatistician, the Statistical and Data Management Center Project Manager, and Leadership and the Operations Center Clinical Research Manager, quarterly or more often, if indicated, and reported to the Medical Officer together with any actions that are taken. Social harms will be summarized and reported to appropriate IRB(s) following IRB guidelines. Any emergency situations will be reported to the parties mentioned above within 24 hours. It is envisaged that refresher training will take place at least six monthly.

**Study monitoring**

On-site study monitoring, both internally (by study staff) and externally (by the DAIDS contractor) will be performed in accordance with DAIDS policies.

**Practical measures to mitigate risk**

In addition to the above strategies, we will implement several practical measures to mitigate risks. These include the following:

1. Identification of the study:

To safeguard participants’ confidentiality, the study will not be identified as a study of MSM. Recruitment materials and messaging for the study will be focused on participation in a health and wellness study. It is the sites intention to conduct this study as it would any of its other studies whilst being mindful of the study population in order to normalize the presence of this study population in the clinic.

1. Screening strategy:

The screening questionnaire will ask men about their sexual behavior with male and female sexual partners in the past 3 months in addition to several other questions. Potential participants will not be asked to self-identify their sexual orientation. In this way, MSM can be selected for participation through the screening questions.

1. Study participation:

The clinic area is on the ground floor of the New Nurses Home at the Chris Hani Baragwanath Academic Hospital. It has a common clinic area as well as other discrete clinic areas on the ground floor that are closely clustered.

The clinic set up allows for flexibility of clinic flow or process dependent on study requirement. Clients are received at the main clinic reception desk at which their visit is registered. The participant medical file is then extracted and the participant is guided to the study consulting room. This is my preferred approach; it applies to all other studies at the Soweto CTU and PHRU.

The HPTN clinic is currently housed in one of the discrete clinic areas. Should there however appear to be problems for the participants, reception and registration of the participants will be possible in the HPTN clinic with minimal disruption to HPTN/PHRU clinic processes.

Privacy and confidentiality as well as respect for persons are one the cornerstone of this research unit that has conducted clinical trials successfully for almost twenty years.

1. Data management:

Detailed locator information, collected at the study Screening Visit and actively reviewed and updated at each subsequent visit, will be stored separately from other study data and kept in double locked cabinets. For each study participant, a unique ID will be generated and assigned; there will be no personal identifiers. Only specific study personnel will have access to any hard copies of data and the computer files.

1. Monitoring staff interactions with participants:

We are dedicated to ensuring the rights and dignity of all study participants. As such, we have taken a number of steps to ensure that staff-participant interaction is in line with our ethos of treating all participants with dignity and respect. The PHRU has been conducting clinical research involving populations that at varying points in time may have been subjected to similar prejudices that MSM now face. Staff are professional in their approach and has developed sensitivity in dealing with vulnerable populations.

Mechanisms to monitor staff-participant interactions may include:

1. A clearly accessible suggestion, comments and complaints box. All participants will be encouraged to provide feedback to study staff via the suggestion box on an anonymous basis. This will enable them to highlight any inappropriate or offensive behavior by staff members, as well as to encourage positive behaviors. In ensuring that we successfully build trust with our target group, staff will meet weekly to discuss comments and brainstorm ways to address them
2. Evaluation forms will be completed after designated study visits. These will ask participants to rate the service they have received, as well as the staff member who assisted them. There will be room for comments should they request.
3. Potential focus group discussions
4. Ad-hoc meetings as problems arise. Meetings may be either formal or informal in nature.

All data will be collected and tracked to identify any trends in the complaints and grievances and will be used to shape any improvements that may be required.

1. Staff’s maintenance of confidentiality:

Staff will sign confidentiality statements at recruitment and on annual basis regarding sharing any participant information, ensuring the privacy of participants. The employment contracts provided to staff of the PHRU addresses the issue of confidentiality of data and information we interact with and collect on a daily basis. Staff orientation programmes on employment stress participant confidentiality and data integrity. Participant and data confidentiality is required by ICH GCP and the protocol. (Section 8.6)

Breaches of confidentiality and disclosure of participant information will be dealt with in line with South African labour law practices and the Wits Health Consortium Human Resource department disciplinary policies. Ongoing review of issues related to confidentiality will happen through the lifespan of the study with refresher training on confidentiality in general as well as specifically to the protocol will occur at a minimum on a six monthly basis.

1. Sensitivity training of CAB:

Health4Men is the lead MSM programme in South Africa, and has built upon MSM research in Soweto and Cape Town to establish MSM targeted services in public sector clinics. This program is currently being expanded to all provinces of South Africa; H4M staff has great experience in working with MSM, in training clinical staff and in providing relevant and acceptable information and education campaigns in the community and in MSM targeted publications.

This site plans to arrange the relevant training through H4M for study staff, CAB members and relevant non-study clinic staff. Refresher trainings and debriefing sessions with the study team and participants will be scheduled. The timing and frequency will be dependent on the nature of the issues that arise.

It is worth noting that the Health4Men Simon Nkoli Men’s Health clinic was based at the PHRU in the past, before moving into a primary health care setting, and existed there without incident. Health4Men’s activities have full support of the Department of Health and local staff, and have been welcomed by the MSM community and clinical staff

1. Complaints about treatment by staff:

South Africa has a National Patients’ Rights Charter that directs both patient and doctor responsibilities as well as the steps patients can take should they not be happy with services they receive from clinicians.(as per the South African constitution and the South African National Health Act)

The PHRU and H4M pride themselves in taking the treatment of participants/patients very seriously. In the event that a participant complains about a staff member, the following will take place:

1. Participant’s complaint will be documented and acknowledged so that participants understand that their complaint will be addressed.
2. Complaint will be brought to the attention of the staff members’ line manager who will
3. Deem the offence minor and discuss the complaint with the staff member
4. Deem the offence major and engage the PI and staff member to discuss the complaint
5. All complaints will be kept on record.
6. Repeat offender will undertake corrective action either through retraining of through the Human Resources department – as appropriate and relevant to the severity of the incident.
7. Outcome of participant complaints will be communicated to the participant and if necessary changes to clinic process or personnel will be effected where reasonable possible.
8. Approach to potential conflict:

The site will strategically to anticipate and resolve potential problems at the site that may affect the conduct of the trial. For example, if MSM are prevented or interfered with when attending the clinic, the following will be our approach:

Pre-study: (one would anticipate that there may be problems and thus plan ahead)

- Sensitivity training for all staff potentially interacting with the participants (including but not limited to hospital security, site cleaning staff, clinic reception and study staff)
- Incident management training
- Non-threatening messaging through strategic placement of posters
- Creation of awareness within the study population of potential problems

During the study:

As above with ongoing assessment of the clinic environment to gauge opinion

Should an incident occur:

- Increase security
- Convene emergency committee
- Investigate the incident and assess scope of the problem
- Create and implement solution in conjunction with the committee, CAB and other relevant stakeholders
- Measure effectiveness of measures implemented (adjust again if necessary)
- Incident reporting to the IEC/IRB/study team

**Risk Mitigation Plan review:**

At a minimum, this risk mitigation plan will be reviewed as part of the site initiation, prior to site activation as well as annually by FHI 360, other study team members, and the site IRB; Site review and amendments may be conducted earlier based on prevailing circumstances, engagement with communities, IRBs, authorities in order to maximize the rights, interests and welfare of participants.

**Details and responsibility in case of general study queries or incidents**

*Note that all media enquiries or requests for interviews must t be directed to Ravindre Panchia, Glenn de Swart or Glenda Gray.*

| **Site Name** | PHRU |
| --- | --- |
| **Site Location** | Soweto, Johannesburg, South Africa |
| **CTU PI** | Glenda Gray |
| **CRS Leader** | Ravindre Panchia  E-mail: [panchiar@phru.co.za](mailto:mkhizeb@phru.co.za); tel. +27 72 125 3175 |
| **Study Coordinator** | Ruth Motlafi  E-mail: [motlafir@phru.co.za](mailto:motlafir@phru.co.za); tel. +27 82 671 9694 |
| **Primary Spokesperson 1** | Ravindre Panchia |
| **Primary Spokesperson 2** | Glenn de Swart |
| **Primary Media Point Person** | Ravindre Panchia  Glenn de Swart |

**Primary responsibility for communicating with and/or coordinating activities aimed at the following.**

| **AUDIENCE** | **Name/position of staff with PRIMARY RESPONSIBILITY** |
| --- | --- |
| **Community Groups** | Nomampondo Barnabas (Community Liaison Manager) +27 83 479 6156  Glenn de Swart |
| **Advocacy & Civil Society Groups** | Nomampondo Barnabas  Glenn de Swart  Ravindre Panchia |
| **NGOs** | Nomampondo Barnabas  Glenn de Swart |
| **Media** | Nomampondo Barnabas  Glenn de Swart |
| **Local Government** | Ravindre Panchia  Glenn de Swart  Glenda Gray |
| **Regulatory Bodies** | Ravindre Panchia  Glenda Gray |
| **Other**  **(Please specify)** |  |

**Details and responsibility in case of emergencies:**

| **Clinic/site security** | Jabu Cindi  Rishlin Naidoo +27  Ravindre Panchia +27 72 125 3175 |
| --- | --- |
| **WHC legal** | JJ du Preez - +27 11 274 9200 |
| **Section 27 legal** | To be named |
|  |  |

**References**

1. Joint United Nations Programme on HIV/AIDS. Good participatory practice. Guidelines for biomedical HIV prevention trials 2011. Geneva: UNAIDS; 2011.
2. Joint United Nations Programme on HIV/AIDS. Ethical considerations in biomedical HIV prevention trials. Geneva: UNAIDS; 2012.
3. amfAR The Foundation for AIDS Research. Respect, protect, fulfill. Best practices guidance in conducting HIV research with gay, bisexual, and other men who have sex with men (MSM) in rights-constrained environments.
4. HIV Prevention Trials Network Ethics Guidance for Research Revised June 10, 2009: Prepared by Stuart Rennie, PhD, MA, University of North Carolina-Chapel Hill; Jeremy Sugarman, MD, MPH, MA, Johns Hopkins University and the HPTN Ethics Working Group.

**APPENDIX 1**

**Questions for Researchers to Ask for MSM/HIV Research**

|  |  |  |
| --- | --- | --- |
| Have you included the MSM community in: |  | *.* |
| Engagement Rules | Yes | The research has been discussed with the collaborating NGO partner and community reactions assessed |
| Situational Assessment | Yes | The research will be linked to the outreach and care services of Health4Men, the MSM service provider in the area. |
| Have you assessed the relevance of the research and potential reactions from greater community structures? | Yes | We consider the opinion of ANOVA Health/H4M as key in representing the interests of MSM. Their involvement in the development of this protocol as well as their willingness to collaborate has given clear indication of interest among MSM. |
| Have you assessed the interest in the MSM community, as well as current infrastructures (or lack thereof | Yes | Health4men has been active in this community for a number of years and has good relationships with many stakeholders in the Soweto community. MSM HIV services and prevention activities are part of the South African National Strategic Plan for HIV and ANOVA’s Health4men program is a lead partner with government services across the country to do this. ANOVA Health is also a member of the South African National AIDS Council’s LGBTI sector, which is the recognized community forum |
| Have you assessed the willingness of your research institution to Respect, Protect and Fulfil rights of participants? | Yes | The PHRU and the ANOVA Health both have a long history of research in this community, and both organisations are covered by the Wits University Human Ethics Committee as their IRB. The institution has a commitment to respect and protect research participants and has approved and monitored many studies in MSM. |
| Have you developed an MOU with community-based organizations – clearly involving them in tall aspects of the research? | In progress | An MOU will be signed between the ANOVA Health Institute’s Health 4Men MSM program and the PHRU once the study is confirmed. Further agreements will be developed in conjunction with the communication plan |
| Have you clearly defined the roles and responsibilities of all stakeholders? | Yes | The roles and responsibilities of PHRU and Health4men have been delineated and will be formalized in the MOU |
| Have you conducted an intensive identification process with stakeholders including: |  |  |
| Community stakeholders, NGOs, CBOs, community groups , informal networks etc. | Yes |  |
| Government ministries, leaders, etc. | No |  |
| Local health care facilities and services | Yes |  |
| Local religious leaders | No |  |
| Media | Yes | ANOVA’s HIV & AIDS Media project provides regular training and informational updates to local media practitioners, which have included a number of MSM related issues. These extensive links to the media can be utilized for this study |
| Have you engaged government, while first discussing effective models of engagement with community representatives? | Yes | ANOVA Health, a key partner for this study current represents MSM on the South African National AIDS Council. (SANAC) The latter organisation is managed by the Deputy President of South Africa. ANOVA Health/H4M also run clinics within the state health sector in collaboration with local and provincial governments. |
| Have you secured funds for community involvement (e.g. providing financial incentives etc.) | Partially | Currently managed through the CAB/community budget. Additional specific funding will be sought in budgeting for this study |
| Will you start by conducting formative research activities to learn more about the target populations and their priorities? | Yes: building on existing research | ANOVA Health and PHRU have conducted several studies in this community in `Soweto since 2008, and a range of other studies in other parts of South Africa, all of which will provide information |
| Have you-included research on human rights protections/violations within the research context? | Yes | We have researched health service attitudes and rights violations and are very aware of research protections. |
| Will you provide research literacy training to key stakeholders? |  |  |
| Local NGOs CBOs, informal networks of MSM |  | ANOVA Health/H4M currently provides a wide range of community education, Specific research literacy can be provided in conjunction with PHRU. ANOVA and collaborating institutions have also provided research literacy and research writing training to government officials in the key populations field |
| Healthcare service providers |  |  |
| Media |  |  |
| Government |  |  |
| Influential community leaders |  |  |
|  |  |  |
| Have you developed policies for dealing with hostile/intrusive media, media that may blame MSM for ‘spreading HIV’ in a country? | Yes | ANOVA Health has a media project and staff who deal with media on a daily basis, and have experience in proactive management of these types of situations |
| Have you developed certificates of confidentiality to help participants feel safe, knowing that their information will not be shared with others? | No | The CTU has been conducting research for years encompassing a large number of protocols. The certificate of confidentiality has not been necessary; This will nevertheless be reviewed with the relevant stakeholders to reach consensus for this study as start. |
| Have you developed personal identifiers that protect people’s identities, or considered conducting research anonymously? | Yes | All participants have unique study identifiers. All personal identifiers are kept in their clinic records separate from the study documentation. Only study staff has access to the information that link personal demographics with the study ID in password protected files. |
| Have you ensured safe storage of any data that would link participants’ sexual orientation information or behavioral practices? | Yes | See note on confidentiality. |
| Have you (or others) conducted formative research activities to learn about: | Yes |  |
| MSM needs and specific priorities | Yes | ANOVA Health has undertaken a range of research in south African settings on these issues, including a recent national report on MSM services and identified community needs in conjunction with the Elton john AIDS Foundation. |
| Prior research in this community | Yes |  |
| Violence/sexual violence | Yes | ANOVA Health and Emory are currently studying IPV in a study in Cape Town, and as part of UCSF collaboration in Mpumalanga. These results will inform work on this study |
| Poverty | Yes | ANOVA’s Ukwazana project has studied some of these aspects |
| Have you planned for MSM/LGBT community capacity-building and informed participation? | Yes |  |
| Secure funding to build capacity of MSM/LGBT community members | Planned |  |
| Allow local groups to use resources such as meeting spaces |  | Already exist for ANOVA Health/H4M. Some existing facilities to be mirrored at the Soweto CTU |
| Ensure representation of MSM/LGBT on staff | Yes |  |
| Train MSM/LGBT community members to be involved as study staff to build capacity for the future | Yes |  |
| In disseminating results, do you have plans to work with MSM/LGBT community leaders on data dissemination and a utilization plan, including media advocacy? | Yes | ANOVA/H4M training and advocacy manager is also the Vice Chair of SANAC, and we will utilise this and other avenues for advocacy |
| Do you plan to build the skills of activists to disseminate/use data locally for advocacy | Yes |  |
